# Promoting smoking and fighting cancer in Australian migrant newspapers, 1930–60

**DOI:** 10.1017/mdh.2026.10064

**Published:** 2026-07

**Authors:** Yianni Cartledge, Andrekos Varnava

**Affiliations:** 1 History Department, College of Human Sciences and Culture, Flinders University, Australia; 2 Faculty of Education, Tabor College, Australia; 3 De Montfort University, United Kingdom

**Keywords:** Australia, cancer, migrant newspapers, migrant press, migration, smoking, tobacco

## Abstract

This paper explores and compares smoking advertisements and anti-smoking and anti-cancer messages in Australia’s migrant press, particularly newspapers, from 1930 to 1960. It investigates the ways in which smoking was promoted to migrant communities through their newspapers, contrasts this with the increasing prevalence of anti-smoking and broader anti-cancer messages, and explores whether there were any shifts in advertising and in anti-smoking messages following the growing research linking smoking and cancer (particularly lung cancer) from 1950. These messages were ultimately tied to this growing research, as well as the various Australian state and national anti-cancer campaign committees which emphasised early diagnosis and swift treatment as the best method to combat a range of cancers. Yet the Australian authorities, although finally acknowledging the dangers of cigarette smoking, rejected any government intervention other than providing the medical reports to the public. Greek-language newspapers (notably *To Ethnico Vema*) form an important case study; however, other foreign-language and migrant community papers were also consulted, including Italian, Jewish, and French.

## Introduction

**Figure 1.**
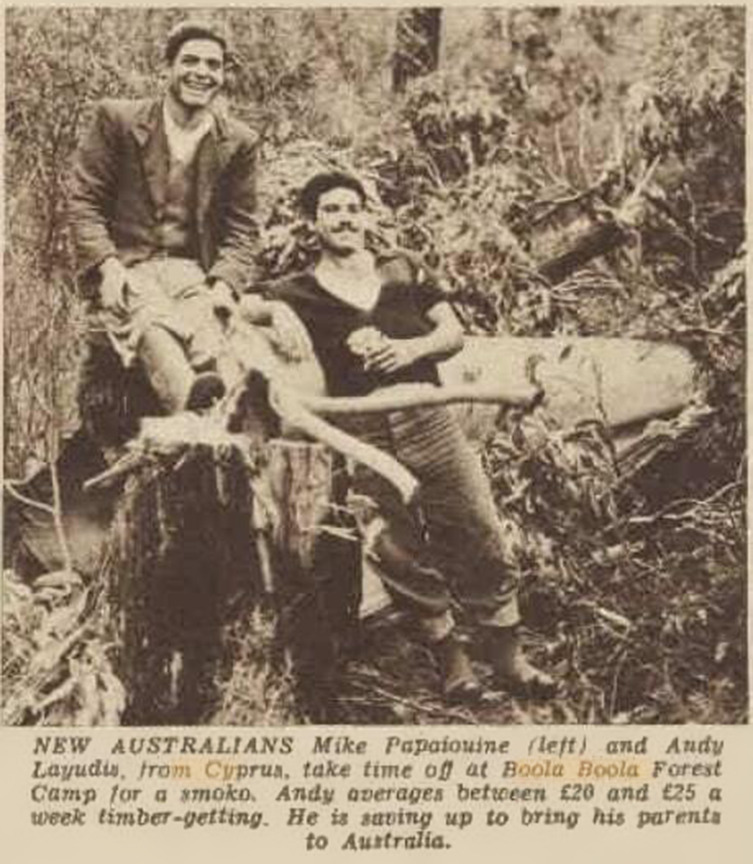
*The Australian Women’s Weekly*, 21 November 1951, 29.

On 21 November 1951, *The Australian Women’s Weekly* featured a story about how Australian gum trees were being used to make paper and in one photograph the ‘New Australians’, Mike (Michalis) Papaioannou and Andy (Andreas) Lavidis, both Cypriots, were shown taking a break from lumberjacking at Boola Boola Forest Camp, in Gippsland, Victoria, for a ‘smoko’ (Figure 1).^1^ A ‘smoko’ is a slang term used mostly in Australia and New Zealand for a short break during work hours, which meant stopping for a smoke. In fact, the ‘smoko’ became an Australian institution, even mentioned in government reports, but as the health hazards of cigarette smoking developed, its meaning was broadened to mean a coffee or tea break. The Cypriots in the photo came from a country, British Colonial Cyprus, with a long tradition and culturally entrenched habit of smoking, and were therefore ‘right at home’ with the concept of the Australian ‘smoko’.^2^

This is the first study to explore both the promotion of smoking and anti-smoking messages to migrants (specifically Mediterranean) during the initial period when smoking was linked to lung cancer. It therefore looks at both the promotion of smoking and the early discussion around its dangers. The first part focusses on the promotional messages and advertising in migrant newspapers in Australia and the second part on the anti-smoking information and messages, which were often within broader anti-cancer campaigns. Since the link between smoking and lung cancer was first made in 1950, and it was not until further reports appeared during the 1950s and early 1960s that the Australian government considered and then took any action, the period under investigation covers 1930–1960. This research seeks to answer several questions around the way that cigarette smoking and anti-smoking messages appeared in migrant newspapers in Australia. The importance of this is evident, as not only are studies on smoking and tobacco in relation to migrants and migrant newspapers scarce but it also helps further advance the study of how tobacco was targeted and advertised to specific cultural groups and subsets within society. Broadly, two questions inform this research: how significant were the promotion and advertising of smoking in migrant newspapers and did this change once the link between smoking and lung cancer was found? And, to what extent did migrant newspapers inform their readers about the links between smoking and lung cancer as these emerged and were debated in public? As a result of exploring these two questions, a third emerged: Were the anti-smoking messages subsumed within broader anti-cancer messages?

Given the questions this study seeks to answer, it is important to understand the context around the discovery of the links between smoking and cancer. In 1950, the UK-based Richard Doll and A. Bradford Hill published a study linking cigarette smoking and lung cancer, which shifted discussion and approaches to the tobacco industry in the Western world.^3^ By 1953, there were already publications warning the British public about the dangers and links between smoking and cancer.^4^ In 1954 E.C. Hammond and D. Horn, a US research team, and a UK medical committee commissioned by Whitehall, confirmed the earlier research and the cigarette-lung cancer link became undeniable.^5^ But many, especially the cigarette manufacturers, denied any link. David T. Cartwright noted, however, that official and policy responses to the new information were gradual, with British and American cigarette companies initially deflecting questions of health, especially during the 1950s.^6^ There were also various scientists aiming to refute the new findings during the 1950s and 1960s, who have since been viewed with ‘a cloud of suspicion and corruption’ by historians.^7^ The press around the cigarette-lung cancer link eventually led to mass corporate denial by the tobacco industry, which included misinformation campaigns lasting into the 1980s, and ‘denialism’ persisting to the present day in many nations.^8^ Further to this, Matthew Hilton noted that after the link was made, culture was also a battle field, and that ‘the medical profession was not competing simply with the defences and protestations of the tobacco manufacturers…but the whole wider promotion of smoking which refused to deny the cigarette’s crucial role in everyday life’.^9^

The real shift occurred after 1962 when the Royal College of Physicians (RCP) in the UK released an expert committee review into smoking and health, which concluded that cigarette smoking was a cause of lung cancer.^10^ But even then, the Australian government delayed action on cigarette control until the 1970s, with health warnings being first mandated on all cigarette packs in 1973, and bans on cigarette advertising on radio and television in 1976. Although historian Ian Tyrrell used 1950 as a turning point, he emphasised that anti-smoking messages in Australia were infrequent during the 1950s, and only shifted slightly during the 1960s, with the mid-1970s seeing the most dramatic changes.^11^ Agnes Toth-Peter et al. echoed this, especially because the 1960s were the peak of cigarette consumption in Australia.^12^ From 1986 to 2006, there were more measures introduced, targeting workplace and public smoking. For example, in 1990, the advertising of tobacco products in newspapers and magazines was banned; a ban which, 2 years later, was expanded to all broadcasting and the publishing of any advertising of tobacco products.^13^

Ultimately, this study bridges the histories of tobacco, migration, newspapers, and public health in Australia from the inter-war to the post-war period. Scholars in these highly developed fields have mainly worked within their silos, rarely linking these distinct, yet interconnected, research areas. The bridge is an Australian one, since the focus is on Australia, and thus this research contributes to the Australian histories of tobacco, migration, newspapers, and public health. This innovative approach to research will contribute to the literature on all these areas, but also aims to move beyond their individualised intellectual frameworks and shift historians from their epistemological anxieties to affirm their links to other fields and to the social and life sciences, especially public health. The research directly links public health in relation to cigarette smoking with the specific dynamics of the migrant groups being studied, because it shows that the relationship of these migrants with cigarettes originated before their migration, mainly through addiction to cigarettes, and for some, through farming tobacco, which continued in Australia.

The article focusses on Mediterranean migrants especially from Italy, Greece, and Cyprus for two reasons. The first, as will be shown presently, because it builds upon more recent work that seeks to expand our knowledge of the early years of the settlement of people from these places. Secondly, and more importantly, because these groups had a connection to tobacco and cigarettes, as large quantities were grown in Italy, Greece, and Cyprus and most were already smokers before arriving, thus, perhaps, making them more susceptible to advertising and to the Australian ‘smoko’. Some also owned tobacco-growing farms in Australia and employed people from their countries and migrants in general. Additionally, Mediterranean peoples, especially males, had higher rates of smoking compared to Australians, as will be discussed.

It is also important to explain why this article focusses on the period before the early 1960s. Firstly, this research is part of a larger project, as the postscript and conclusion hint to further research on the period after the 1960s. Therefore, this article aims to provide an understanding of the earlier history of promoting smoking and anti-smoking information during the period of the 1950s when the links between smoking and cancer were known and countries were starting to provide public health advice and take measures. The period before 1950 is important in understanding the promotion of smoking and how strong this was, especially within migrant newspapers, and necessary to understand any continuities and discontinuities with promotion of smoking in the 1950s.

## Historiography and methodology

There have been significant studies on global tobacco, especially in the Anglosphere. These studies have laid a foundational understanding of the origins, changes, and policies, surrounding tobacco and cigarette control, as well as on cigarettes in popular culture. Iain Gately’s popular account of the cultural history of tobacco showed, via a long history of the plant, how it came to spread throughout the centuries, and transition from an occasion practice smoked in a pipe, to the highly industrialised and commercialised cigarette, with its cultural domination in the twentieth century.^14^ In another approach, Howard Cox’s exploration of international cigarette networks, particularly the British and American tobacco industries (dominated by the British American Tobacco company) from 1880 to 1945, emphasised the global nature and imperial dimensions of tobacco and cigarettes, and the shifting nature of the market, which thrusted tobacco companies into global multinational organisations.^15^ Cox’s study worked in partnership with Matthew Hilton’s examination of smoking in British popular culture in the nineteenth and twentieth centuries. Hilton argued that smoking was portrayed as sophisticated and erotic, and was central to masculine and feminine identity and camaraderie in Britain and the wider British world.^16^ In Australia, Ian Tyrrell provided a comprehensive historical overview, using 1950 as a ‘turning point’, though the first ten chapters are on the period before 1950 and only the last four on the period after. Tyrrell showed how the tobacco industry expanded significantly in Australia in the mid-1950s, as if to counter the growing medical evidence linking it to lung cancer and other emerging health concerns. He also showed that the anti-smoking messages shifted only slightly after 1950, improving only after further evidence in the early 1960s confirmed the link, and it was not until the mid-1970s that they became stronger.^17^ During the 1980s, historians began analysing and highlighting the importance of anti-smoking messages in media, foreseeing it as a key component in reducing cigarette usage long-term.^18^

The histories of migration to Australia are vast, so there is no real start or end point, since research continues given the significance of migration to Australia’s past, present, and future. The most significant general works have established that the post-war period saw the arrival of more migrants than at any time in Australia’s history, with preference for British people from the UK, though from the early 1950s the numbers each year ebbed and flowed.^19^ Assisted passage agreements with countries such as Malta, Italy, Greece, and Yugoslavia facilitated the movement, yet also controlled the numbers, as the Australian authorities set the quota each year. Most recent research has shown that certain groups, such as Cypriots, were restricted, beyond the restrictions associated with well-known ‘white Australia’ policy because they were deemed ‘undesirable’ and ‘suspect’.^20^ The ‘white-Australia’ policy, which restricted non-Europeans based on their appearance, finally ended in the 1970s. Most importantly for this project, given its methodology, is the recent focus on migrant newspapers and how important these are to the research into migrant communities in Australia and how much more there needs to be done using these sources.^21^

There have been some studies on cigarette smoking and cancer among Mediterranean migrants in Australia, but these have been on a later period than the one under investigation here. This has included the exploration of the Greek community in Sydney and whether the ‘Good Heart, Good Life’ campaign during the 1990s, which utilised Greek-language anti-smoking media, had significant results – which it did, to an extent.^22^ This is comparable to anti-smoking campaigns in Greece between the 1970s and 1990s, which found that systematic anti-smoking messages and advertising campaigns were more effective than the ban on cigarette advertising.^23^ Additionally, in 1997, Lisa Trotter compared the smoking beliefs and behaviours of Greek and Chinese communities in Australia, who both had higher smoking rates than their locally born Australian counterparts, ‘due to the retention of cultural norms’, as well as the Quit campaign targeted at them, which was found to have been mildly successful.^24^ Similarly, there has been a study on the socio-cultural influences on older Greek Australian smokers, which concluded that cigarette ‘smoking has been accepted as a social and cultural norm’.^25^ Prior to these studies, in 1995, Myriam Khlat investigated Mediterranean migrants (mostly from North Africa, the Near East, and Italy) in both France and Australia, and found that they had lower rates of certain cancers (including lung cancer) than locally born populations (despite higher levels of cigarette use), which she put down to the benefits of the Mediterranean diet and genetics (especially for Moroccans), among other factors.^26^

There is a growing interest in public health histories that moves beyond the simple retelling of successful medical developments and explores social, economic, political, and cultural aspects of the history of public health. The purpose of this article is to contribute to one specific aspect of public health history, that is, the anti-smoking messages following the link between smoking cigarettes and lung cancer in 1950. Since the 1990s, American, British, and Australian academics have reflected on certain aspects of anti-smoking measures and tobacco control, to evaluate whether the changes in the second half of the twentieth century had been successful and what was further needed for the future.^27^ Some of these discussions have been enveloped by broader studies on anti-cancer campaigns, such as by David Cantor, Stephen Snelders et al., Elizabeth Toon, and others.^28^ More recently, however, studies have shifted their focus to the societal changes and tobacco habits caused by anti-smoking campaigns and advertising (in Australia, the West, and internationally more broadly)^29^; while also building on the groundwork laid by earlier historians of tobacco control and anti-smoking campaigns, such as Kenneth Warner, and John Pierce et al.^30^ Comparatively, the significant studies on tobacco control, which link the Anglosphere, global networks, and national, socio-cultural, and localised approaches, such as by Robert Proctor, Sarah Milov, Tyrrell, and others, form important context for this research.^31^

This study will contribute to and bridge the fields outlined above, while also confirming the previous academic position that acknowledges the slow acceptance of Doll and Hill’s findings, and thus slow action towards change. While there is general consensus that 1950 was the turning point that brought about the shift towards anti-smoking messages, this study brings this ‘before and after’ into doubt. It seems more apt to say that the 1950 findings were when the battle lines were drawn and a long process began to implement anti-smoking measures. However, at this point, the pro-smoking camp still had the upper hand, as they kicked into action to combat the science and adapt their marketing, while also expanding their economic, social, and cultural significance in Australia’s society – as they did elsewhere. It appears more likely, and this is for a future study to show, that the swifter turning point comes with the further confirmation and mounting evidence of the link in the early 1960s, which finally led to more meaningful anti-smoking measures in the 1970s. The new observations made are in relation to how migrants were featured in this debate. Tobacco companies targeted migrants because many already had a strong smoking habit from their old countries; they primarily worked in factories or as labourers, which was conducive to having the ‘smoko’, and consequently they willingly accepted the ‘smoko’. Additionally, they wanted to be accepted into Australian society. Finally, they were targeted because many lacked a formal education beyond primary school and therefore could not understand the complexities of the debate that emerged, particularly as it was two-sided and confusing to disentangle the science from the promotion of smoking, which was so engrained in their culture, as well as being addictive.

The methodology adopted is that of archival interrogation to unravel, contrast, and compare the ways in which migrant community newspapers promoted smoking and informed their readerships about anti-cancer and anti-smoking messages. This archive-centred approach aims to foster an analytical outlook, especially as this is a novel research topic without a clear seminal work to follow closely. Newspapers are an underrated and sometimes even devalued source for historians, but this study shows how important they can be to reconstructing the historical record. Migrant newspapers (with a focus on the Mediterranean socio-cultural region) were accessed and translated by the authors, most notably the Greek-language newspaper *To Ethnico Vema* (*The National Tribune*, covering 1931–54), and to a lesser extent *Eleftheri Phoni* (*Free Voice*, 1956-57); the Italian language *Il Risveglio* (*The Awakening*, 1944–54), *Stampa Italiana* (*Italian Press*, 1931–32), *Il Giornale Italiano* (*Italian Newspaper*, 1932–40), *Il Canguro* (*The Kangaroo*, 1955–57), and *Eco Italiano* (*Italian Echo*, 1958–59); a number of English-language Jewish newspapers, the *Hebrew Standard of Australasia* (1895–present), the *Australian Jewish News* (1935–present), *Sydney Jewish News* (1939–54), *Australian Jewish Herald* (1935–68), and the *Jewish Weekly News* (1933–35), and the French-language *Le Courrier Australien* (*Australian Courier*, 1892–2011). Together these represent a serious collection of migrant newspapers.^32^

To interrogate these newspapers, keyword searches were employed in various languages translatable to the authors (English, Greek, Italian, and French) via the National Library of Australia’s Trove platform – most notably the terms: ‘tobacco’, ‘smoking’, ‘smoke’, ‘cigarette’, and ‘cancer’. Additionally, newspapers’ advertisement sections were explored manually due to not consistently appearing in searches. In all, around one hundred articles were closely examined across thirteen newspapers, published between the 1930s and 1950s. These one hundred were selected from a much larger number (in the vicinity of around 400–500) as relevant to this study due to showing clear and intentional messaging aimed at migrants. These were also paired with other primary sources, which feature in this article.

This study therefore adds further to the recent work that uses migrant and minority newspapers in Australia, namely the two volumes by Catherine Dewhirst and Richard Scully, which explored the transnational and challenging voices in Australia’s migrant and minority media.^33^ Particularly relevant to this study are the chapters on the political, social, and cultural attitudes in foreign-language newspapers, such as the Italian-language papers *L’Italo-Australiano*, *L’Italiano*, *La Fiamma*, *Il Globo*, and *Nuovo Paese*, Spanish-language newspaper *El Expreso*, Jewish magazines, and the French-language paper *Le Courrier Australien*, which this study also utilises.^34^ Other contemporary sources were also examined. Materials produced for migrants, including English-language workbooks and radio broadcasts, were also accessed and used. Additionally, official records of the National Archives of Australia were also utilised, even if sparingly, which points to the fact that the government did not begin to explore the introduction of anti-smoking measures until the 1960s.

## The newspapers

Migrant newspapers reflected diverse perspectives from within their respective diasporas. They also reflected the views of their owners and editors, and what they also perceived to be the views of their readerships. These are important points to keep in mind when using newspapers as much as this study has done. What follows is therefore a brief background to some of the newspapers that feature prominently in this research. However, before discussing the newspapers, it is important to establish the case that large numbers of Italians, French, and Greeks, as well as Cypriots and Egyptians, many of whom would read Greek-language newspapers, were arriving in Australia from the late 1940s.^35^ According to the censuses, the population born in Italy, Greece, Cyprus, and Egypt grew substantially from the censuses 1933 to 1961, as the following table shows. Additionally, between 1933 and 1961, Australia’s Jewish population rose from 23,000 to nearly 60,000, especially due to the persecution by Nazi Germany before, during, and after the Second World War (Table 1).^36^

**Table 1.** Australian census results by birthplace, 1933–66: Relevant Mediterranean cases^39^ Table 1. long description.

Country/Colony	1933	1947	1954	1961
Italy No.	26,756	33,632	119,897	228,296
Greece No.	8,337	12,291	25,862	77,333
France No.	2,587	2,215	4,699	5,409
Egypt No.	561	803	8,150	16,287
Cyprus No.	502	681	5,773	8,576
Jewish (by religion)	23,555	32,019	48,436	59,329

The Greek language newspaper *To Ethnico Vema* (*The National Tribune*), which is widely referenced in this article, was launched between 1912 and 1913, originally in Melbourne under the title *Australis* (or, *Afstralia*).^37^ Around 1922, the paper was bought by Nikolaos Marinakis (whose brother was the priest Dimitrios Marinakis, who also became an editor) who transferred it to Arncliffe, Sydney, NSW, where it was eventually edited by P.T. Aroney (originally Aronis), a member of the early Greek migrant families of Sydney.^38^ The paper was noted as politically neutral, although it was a staunch supporter of the Greek Orthodox Church and contemporary historians have considered it conservative.^40^ Similarly, *Eleftheri Phoni* (*Free Voice*), the 1956–57 Perth-based Greek-language newspaper, edited by M. Vlahopoulos and C. Dailakis, aimed to be ‘unbiased and genuine’.^41^ Before its final editions in 1996, *To Ethnico Vema* was the second-oldest foreign-language newspaper in Australia, behind French-language *Le Courrier Australien.* ^42^ *Le Courrier Australien* had been launched in 1892 in Sydney by Polish man Charles Wroblewski (who had also launched the German-language paper *Deutsch-Australische Post* in 1893), and ran for 119 years until 2011, with the Mauritian migrant Leon Magrin serving as one of its early editors.^43^ The longest serving editor was M. Albert Sourdin, a Free France supporter during the Second World War, who held the editor position from 1940 to 73.^44^

Australia’s Jewish newspapers, mostly published in English, were similarly concentrated in the large centres of Sydney and Melbourne, with the earliest being the *Hebrew Standard of Australasia* (currently known as the *Australian Jewish News*) founded in Darlinghurst, Sydney in 1895 by journalist and anti-Zionist Alfred Harris.^45^ It was followed by the Melbourne-based 1935 *Australian Jewish News* owned by Leslie (Lazar) Rubinstein (who originally founded the *Jewish Weekly News* after purchasing the 1931–33 Yiddish-language paper *Oystralyer Lebn*/*Australier Leben*/*Australian Life*).^46^ The *Hebrew Standard* and *Australian Jewish News* merged in 1987.^47^ These papers were complemented by the *Australian Jewish Herald* (1935–68), published by Andersons’ Printing & Publishing Co. in South Melbourne, which was known as the ‘official organ of the Jewish community’; and the *Sydney Jewish News* (which later became the *Australian Jewish Times*), from 1939 to 54, which was also published in Yiddish until 1940.^48^

Similarly, originating during the Second World War period, *Il Risveglio* (*The Awakening*, 1944–56), the Sydney-based Italian-language paper, was published by the Australian branch of the Italian anti-fascist movement *Italia Libera.* ^49^ *Il Risveglio* opposed pro-fascist papers, like Perth chemist Luigi Mistrorigo’s 1931–32 *Stampa Italiana* (*Italian Press*) and Franco Battistessa’s 1932–40 *Il Giornale Italiano* (*Italian Newspaper*, which also had an English section).^50^ The short-lived post-WWII Perth newspapers, *Il Canguro* (*The Kangaroo*, 1955–57), founded by Calabrian Australian diaspora figure Alfredo Strano,^51^ and *Eco Italiano* (*Italian Echo*, 1958–59), produced and edited by Armando Raspa, claimed to be non-political.^52^

## Migrants, labour, and the tobacco industry

During the inter-war years, migrants from Italy, Greece, Yugoslavia, and Bulgaria played a pivotal role in the tobacco industry, especially in production, and this led the industry to call for more migrants, especially from Greece, in the late 1940s and early 1950s. In Western Australia most of these migrants worked on tobacco farms, including that owned by the Michelides family, which had been started by brothers Peter and Michael Michelides in 1905, and had succeeded in bringing their families over to also work in the industry.^53^ In Queensland, there were numerous tobacco growers who came from Italy and to a lesser extent Yugoslavia, who employed migrants from Italy, Malta, Yugoslavia, and Greece. One notable example was Italian migrant, Osvaldo Bonutto, who was integral in establishing North Queensland’s tobacco industry, which was explored in his autobiography, *A Migrant’s Story.* ^54^

From 1948 various government and industry figures called for government assistance to facilitate migration from Southern Europe to increase the labour in the industry. In June 1948 J.M. Allan, the Officer-in-Charge of the Tobacco Industry in the Department of Agriculture in Western Australia suggested that the labour shortage in the tobacco industry could be solved by the government finding a means to assist migrants from Greece to work in the industry. T.H.E. Heyes, the secretary in the Immigration Department, responded that the government was in no position to adopt this policy and that any assisted passage agreement would need to apply to migrants more generally from the same country.^55^ In September H.W. Chaffey, the national ‘Tobacco Adviser’, who had visited Western Australia, made an almost identical request to that of Allan, to which Heyes replied as he did before.^56^

In 1950 the proposal to link immigration with the skilled labour shortage in the tobacco industry was taken up on a nation-wide level. At the 33rd meeting of the Standing Committee on Agriculture held in June 1950 the committee recommended that

…the Immigration Authorities be requested to examine the possibility of obtaining, from Southern Europe, migrants of suitable types experienced in tobacco leaf production, with a view to their being engaged in tobacco production in suitable areas in Australia.^57^

Immigration Department secretary Heyes replied that before seeking such migrants from Italy and Greece, a search of those recently arrived from Yugoslavia, Poland, Hungry and other tobacco-growing countries should be made with any qualifications or experience.^58^ The Department of Labour and National Service reported that there were twelve men with experience at Bonegilla (a post-war migrant camp), six had practical experience in tobacco cultivation, three had theoretical qualifications, having undertaken courses in tobacco growing at agricultural schools, and three others had worked in cigarette manufacturing as blenders, graders, moisture testers, and foremen.^59^ While these men were completing their employment at Bonegilla, Heyes was looking into bringing more skilled migrants from Italy as part of their assisted passage agreement. At the same time, the Queensland government revealed a plan to establish 800 tobacco farms and that it would need migrants to work on these.^60^ The Director of the War Service Land Settlement Division, R.W. Wilson, replied that initially the farms would be allocated to returning ex-servicemen, but that there would be potential later for land to be made available for migrants, while labour from migrants would likely also be necessary.^61^

Lists of registered tobacco leaf growers in Queensland from 1952 to 1956 reveal a tremendous number of migrants owning farms. Of the about 700 farms, around 250 had migrant names, with around 200 of Italian descent, the rest being Yugoslav, with smaller numbers of Greeks, and Cypriots, including Turkish Cypriots.^62^ These lists indicate that a substantial number of tobacco growers emerged after the links between cancer and cigarette smoking had been made, and that there was disproportionate ownership by migrants, who employed many workers from their own ‘home’ countries. Migrants from Southeastern Europe thus played an important part in Australia’s tobacco production because of their prior experiences in Europe.

## Promoting the cigarette

From the 1930s through to the 1950s cigarette smoking rates remained high in Australia. Although Tyrrell only provided estimates, these show that from 1900 to 1945 at least 70% of men smoked cigarettes. Women, who had low rates of cigarette smoking before the Second World War, were estimated to have been 26% of cigarette smokers in 1945. While the percentage of female smokers increased to around 29% by 1962, men dropped to 57% from the late-1950s.^63^ This indicates that by 1962 the cigarette smoking rates in Australia were still high at around 43% of the population. These levels were kept high in part because of the prevalence of smoking by Southeastern European and Mediterranean migrants, who were coming from places with higher smoking rates. This includes Italy, where smoking rates increased between the 1930s and 1950s, before declining, with around 64% of men aged between 20 and 24 smoking in 1955, and 70% of men aged between 25 and 29 smoking in 1960.^64^ Additionally, the Eastern Mediterranean, including Greece (where at least 64% of males smoked in 1961) and Cyprus, had similarly high rates, which remained from the 1950s until at least the early 2000s.^65^ Additionally, Greek smokers were smoking on average between 8 and 10 cigarettes daily, and Cypriots were smoking 10 or more per day even in the 1980s.^66^

**Figure 2.**
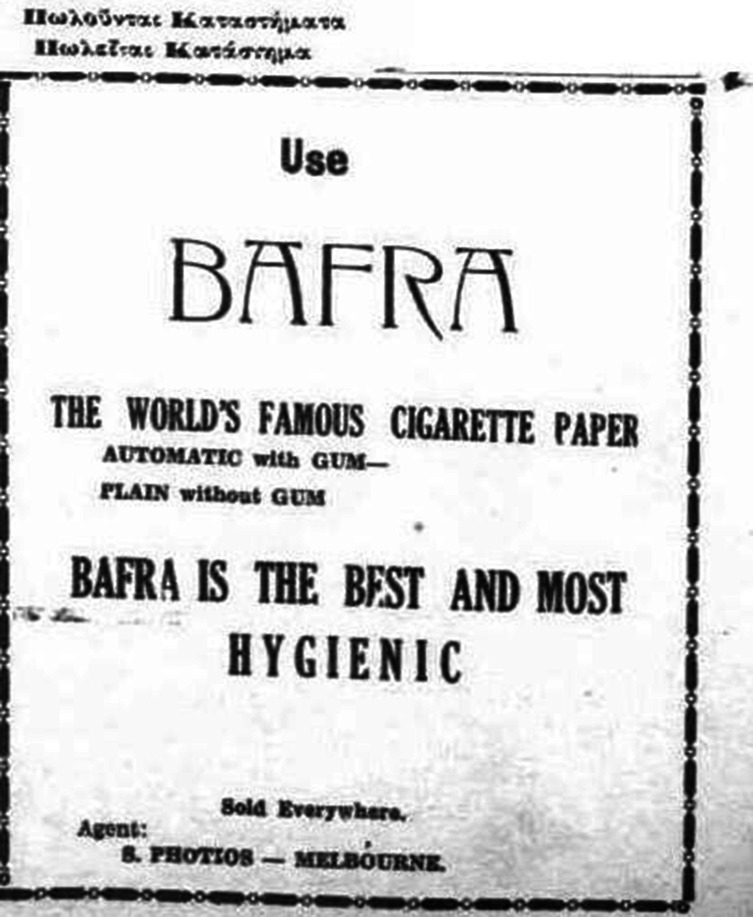
Bafra Cigarette Paper, in ‘Advertising’, *To Ethnico Vema*, Arncliffe NSW, 3 February 1937, 4.

**Figure 3.**
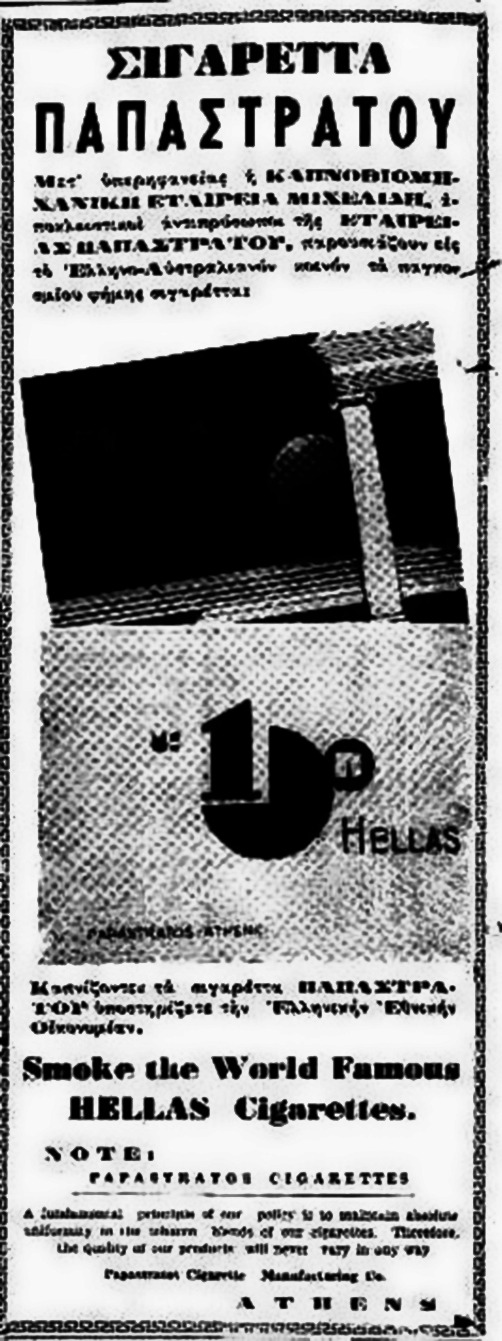
Papastratos Cigarettes, in ‘Advertising’, *To Ethnico Vema*, Arncliffe NSW, 22 June 1949, 4. Figure 3. long description.

**Figure 4.**
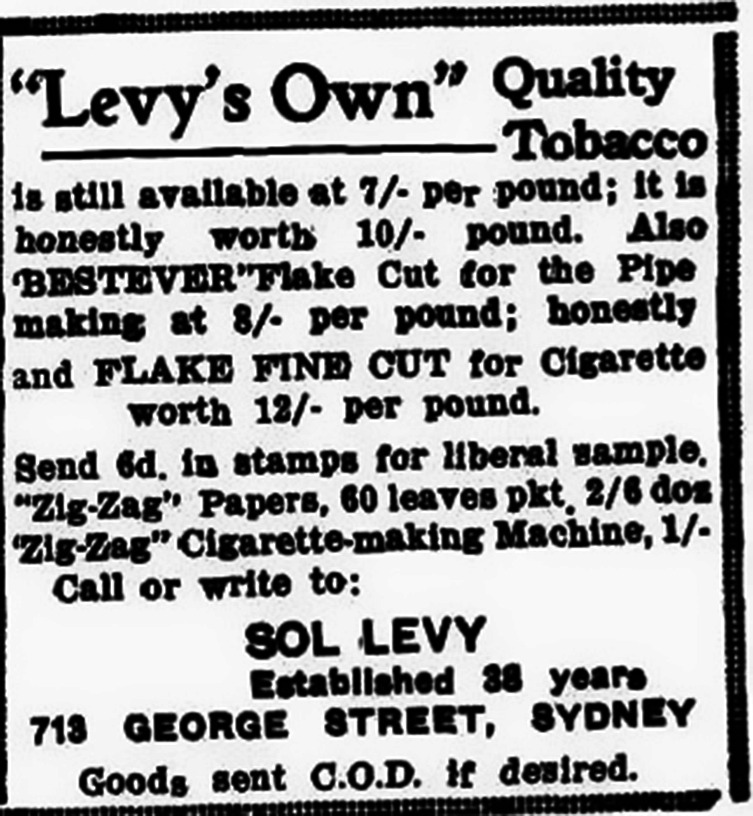
‘“Levy’s Own” Quality Tobacco’, *Hebrew Standard of Australasia*, Sydney, 3 October 1940, 5. Figure 4. long description.

**Figure 5.**
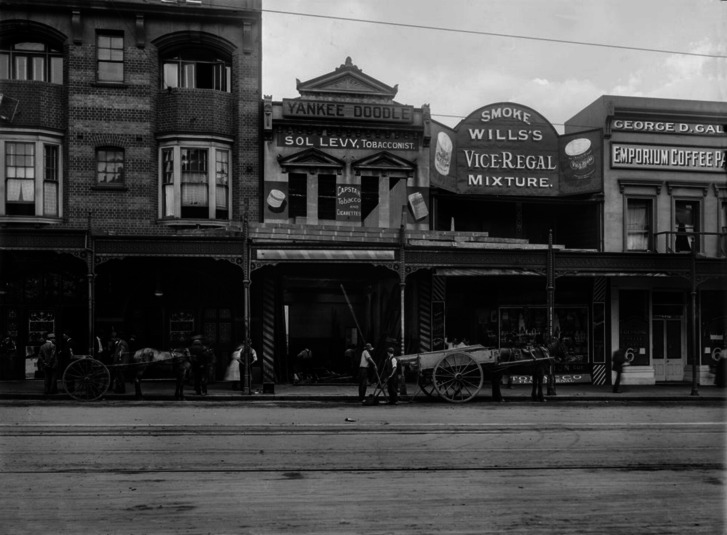
Sol Levy Tobacconist, George St, Sydney, 1912.^73^ Figure 5. long description.

**Figure 6.**
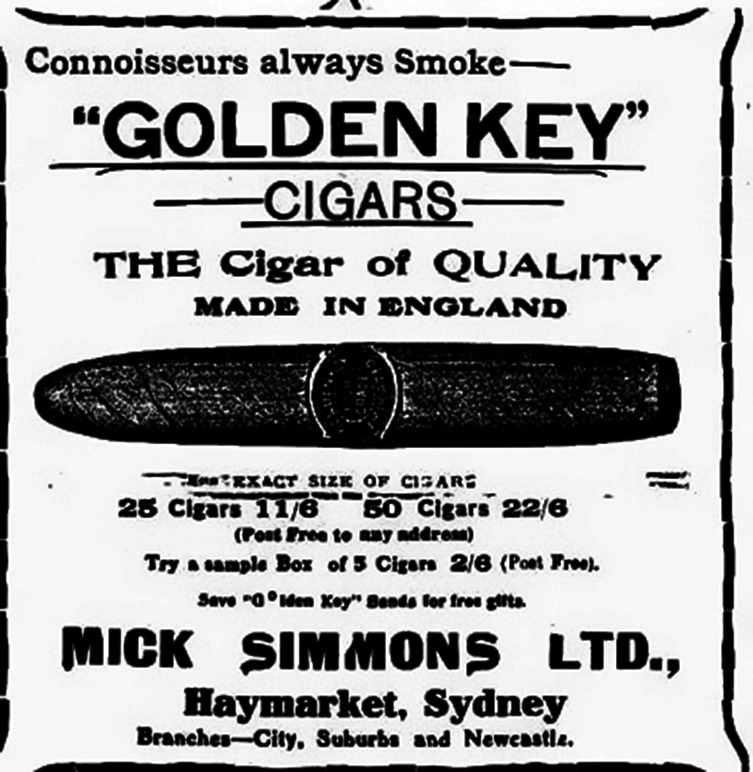
‘Golden Key Cigars’, *Hebrew Standard of Australasia*, Sydney, 15 October 1915, 12.

Cigarette smoking was promoted in the press. Advertisements and an interest in tobacco production were featured in the Greek-language newspaper *To Ethnico Vema*, especially during the 1930s–40s. The promotion of cigarette and cigarette paper brands regularly appeared in newspapers, such as Bafra Cigarette Paper, which claimed to be the ‘world famous’ and ‘the best and most hygienic’, and were imported by S. Photios, Melbourne (Figure 2).^67^ Greek cigarette brands were also featured, including the also ‘world famous’ Papastratos Cigarettes (Figure 3), advertised in 1949 and into the 1950s.^68^ In 1933, Kookaburra Cigarette Paper Tubes were advertised and encouraged in the *Hebrew Standard of Australasia*, which claimed ‘No nicotine will get to your lungs and no tobacco will enter your mouth – they do not stain your fingers and prevents cigarette cough’, hinting at early fears around the dangers of smoking.^69^ Local tobacconists were also featured in community papers, such as Lexington Tobacco Co. of William St, Melbourne, in the *Australian Jewish News* in 1951, who were the ‘Importers of the famous Lexington Cigarettes, made in Luxembourg & Ritmeester Cigars, made in Holland’,^70^ and Levy’s Own Quality Tobacco, owned by Sol Levy of 713 George Street, Sydney, established in 1890 and closed in 2014, and advertised in the *Hebrew Standard* in 1940 (Figures 4 & 5).^71^ Cigars and cigarettes had appeared in Jewish newspapers, such as ‘Golden Key Cigars’, featured in the *Hebrew Standard* since 1915 (Figure 6).^72^ In this sense, migrant newspapers, as much as non-migrant ones, relied on tobacco advertising during this period, with the difference being that brands from the ‘old countries’ were also featured, as the nostalgia of smoking those cigarettes may have reminded smokers of home.

**Figure 7.**
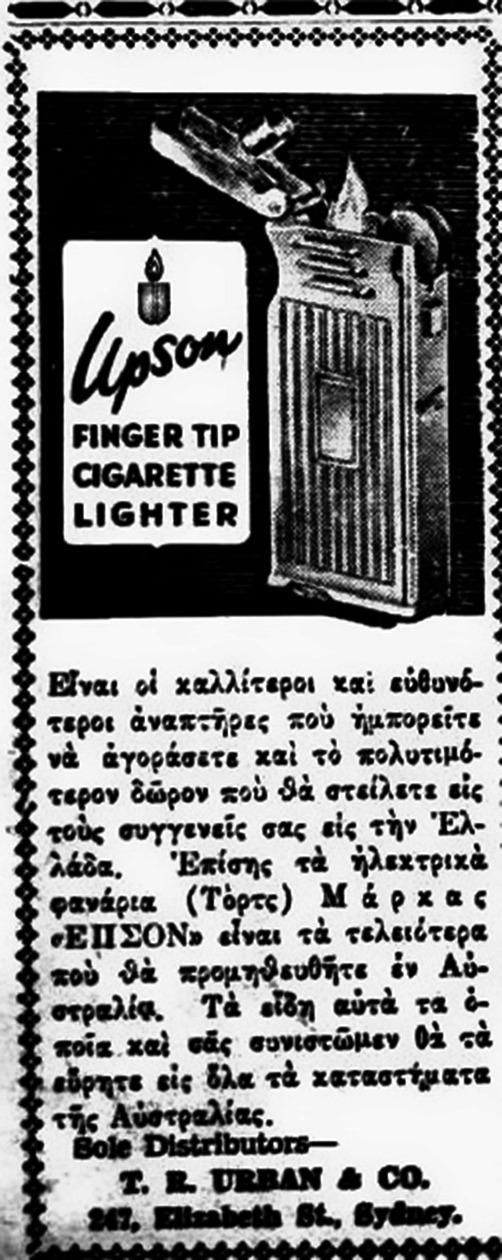
Upson Finger Tip Cigarette Lighter, in ‘Advertising’, *To Ethnico Vema*, Arncliffe NSW, 1 February 1950, 8. Figure 7. long description.

**Figure 8.**
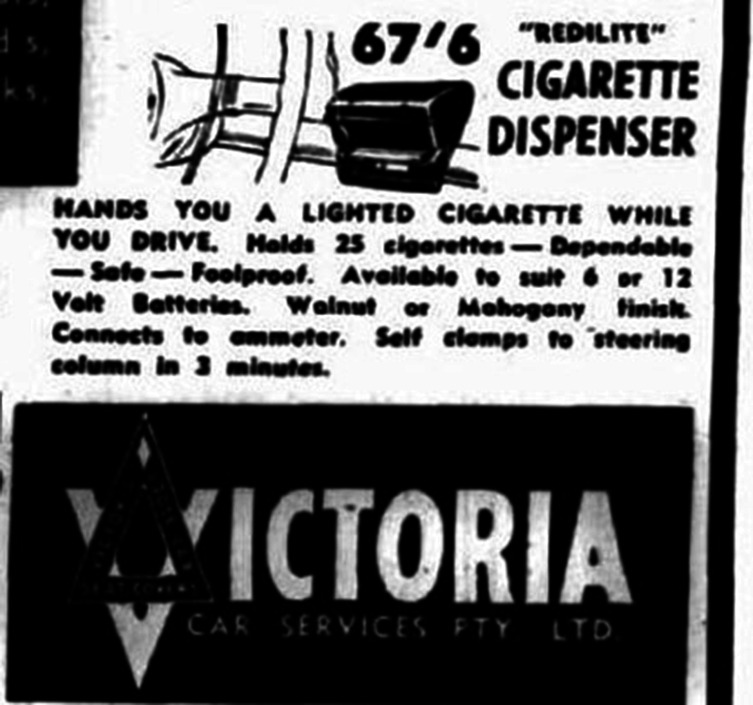
Redilite Cigarette Dispenser for cars, in ‘Advertising’, *Australian Jewish News*, Melbourne, 6 May 1955, 9. Figure 8. long description.

**Figure 9.**
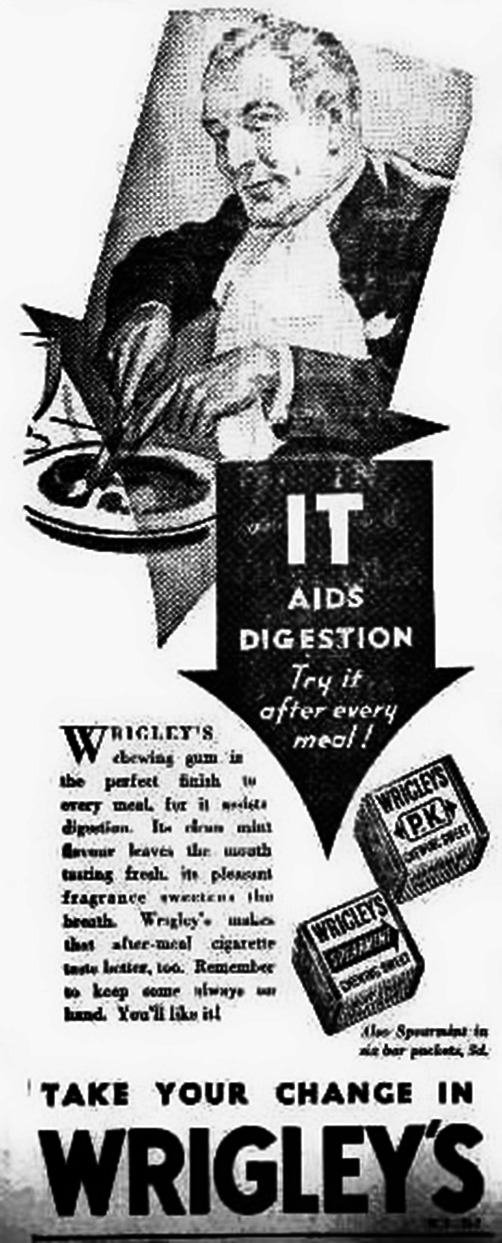
Wrigley’s Chewing Gum, in ‘Advertising’, *To Ethnico Vema*, Arncliffe NSW, 29 May 1935, 2. Figure 9. long description.

Additionally, Australian companies made sure to advertise their smoking paraphernalia. In 1950 the Sydney-based Upson Finger Tip Cigarette Lighters (Figure 7) featured in *To Ethnico Vema*,^74^ and in the *Australian Jewish News*, the Redilite Cigarette Dispensers for cars in 1955 (Figure 8), which ‘hands you a lighted cigarette while you drive’.^75^ Even Wrigley’s Chewing Gum capitalised on this era of tobacco advertisement and migrant targeting (Figure 9), noting that ‘Wrigley’s makes that after-meal cigarette taste better, too’.^76^ Tobacco production was also discussed. In 1939, it was noted that Greece was pushing itself into the world’s tobacco market, with Britain taking an interest in Greek tobacco brands.^77^ In 1948, it was boasted that Greece had imported 242,535 kgs of cigarette paper in the first quarter of 1947, a number that had increased fourfold over the last decade, despite Greece also being a producer of cigarette paper.^78^ In 1947, the expenditure for a Greek community fete was published in *To Ethnico Vema*, which included 9 shillings worth of Greek cigarettes supplied.^79^ These discussions hinted at a cultural interest in tobacco production amongst the Greek Australian community as well as a keen interest in smoking Greek brands.

Smoking was not just advertised in news circulars and paid advertisements, but also in cultural articles and information for prospective and newly arrived migrants. This included one front-page article in 1935 that explored the cigarette’s origins, tracing it to an apocryphal Egyptian soldier in Syria in 1832, who lost his pipe and decided to roll tobacco in a corn husk leaf.^80^ Transcripts of ‘New Australian’ radio broadcasts which taught English to Greek migrants, especially during the 1950s, also promoted smoking-related language and culture:

Is John smoking a cigarette?

Yes, he is smoking a cigarette.^81^

It is a cigarette. It is a match…

This cigarette is here. This match is here…

That cigarette is there. That match is there.^82^

This was similar to an article 20-years earlier, in 1934, which described the Australian ‘beer lover’s order’ as being ‘A packet of cigarettes and three glasses of beer’, which presented itself as ‘cultural lore’ and an important lesson for Greek Australians.^83^ Even the very first lesson (Lesson 1, Part A) of the Commonwealth government’s compulsory language workbook *English for Newcomers to Australia* (1948), included the word ‘cigarette’ as essential vocabulary, among only eight other nouns.^84^ An article, titled ‘Migrants’ tuition “practical”’, published in Adelaide’s *News* in 1950 read that ‘The price of cigarettes, and reading of a railway timetable were among the first exercises in English courses for New Australians’.^85^ This was criticised, not because of the content, but because it was taught ‘in a dull manner’ and did not include grammar – to which an education spokesman defended as the ‘direct method’ and conversational English. This reflected the fact that as part of integrating into Australian culture, prospective and newly arrived migrants should be aware of the significance of and were invited to partake in the Australian ‘smoko’, like the Cypriots discussed at the start of this article.

During the war years, there was seemingly an increase in the promotion of smoking for its health benefits, which was approved by the health professionals and government in order to prosecute the war. The government created the Tobacco Distribution Committee to facilitate the distribution of cigarettes and tobacco. There were concentrated efforts to supply cigarettes to various Australian forces in Australia and abroad, from the Australian Imperial Force, the Royal Australian Air Force, the Royal Australian Navy and the Australian Women’s Land Army, and other units, as well as POWs.^86^ In 1944, when bushfires raged across Victoria and killed fifty-one people, the Tobacco Distribution Committee ensured that cigarettes were supplied to areas impacted.^87^ In 1944 a large quantity of tobacco and cigarettes, provided by the German Red Cross to POWs at Love Day Camp, was stolen and sold on the black market.^88^

In the post-war period, when suspicion started to fall on cigarettes being the cause for the increase in many ailments, especially lung cancer, newspapers acted as apologists for cigarettes. In *Il Risveglio* in 1946 one article read:

**Can’t have cigarettes?**

No! Contact your doctor or some hospital and you will immediately have a prescription (that’s right) that allows you to have, at least, a box of 20 packs of cigarettes. Safe? Very safe. Here it is: W. W. Lupton, who runs a pharmacy in Onawa, Iowa, was presented with a customer with a prescription issued to him by Iowa Hospital for a box of 20 packs of cigarettes. The pharmacist filled the prescription and wrote on the box: ‘Tobacco cigarettes 20 — Use as Directed’. Comments? No. The reader who can’t have a delicious smoke will do them.^89^

For the Italian community, tobacco was additionally an important source of employment in Australia during the 1940s, with job advertisements being frequently posted in migrant newspapers.^90^ For example, one 70-acre tobacco farm in Yelarbon, Queensland, which was being sold by Peter Bonutto (a nephew of Osvaldo Bonutto),^91^ was advertised in the *Il Risveglio* in 1947, and noted as ‘Excellent soil for cultivation of tobacco’.^92^ Advertisements like this also came alongside calls for unionising the tobacco industry, especially to improve the working conditions of Italian migrant workers,^93^ the creation of tobacco cooperatives,^94^ as well as protests due to an increase in the tobacco price, which did not benefit the farmers.^95^ It has also been established that Italian workers in Australia had significant rates of smoking, with a minimum of 40% of all migrants between 1943 and 66, although this was in line with the general population’s statistics.^96^ The cultural links of tobacco remained strong in the Italian Australian community in the late 1940s, with 300 cigarettes being given as third prize in the Australian Relief for Italy committee’s lottery in Melbourne.^97^

**Figure 10.**
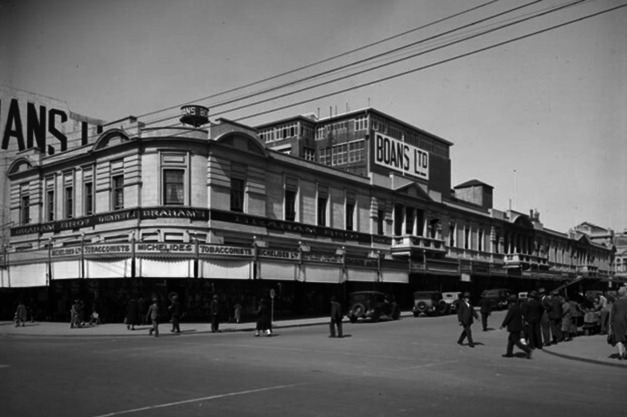
Michelides Tobacconists shop once occupied a central corner in Forrest Place, 1951 (Supplied: State Library of Western Australia): https://www.abc.net.au/news/2020-02-09/perths-once-thriving-tobacco-industry/11924478 Figure 10. long description.

**Figure 11.**
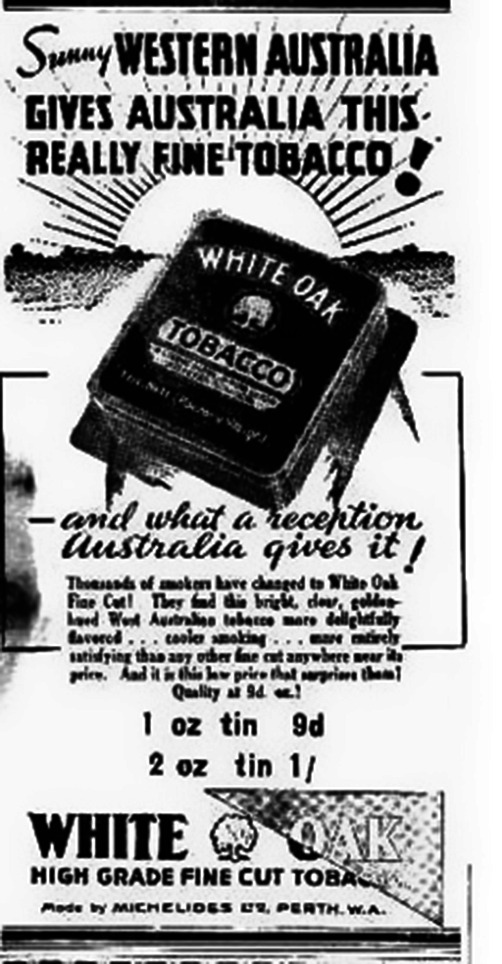
White Oak, Michelides Ltd, in ‘Advertising’, *To Ethnico Vema*, Arncliffe NSW, 3 June 1936, 7. Figure 11. long description.

**Figure 12.**
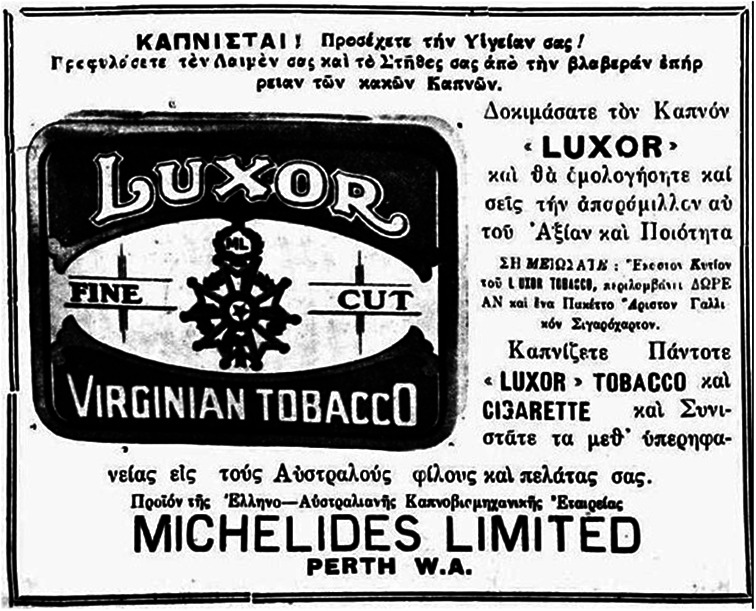
Luxor Virginian Tobacco, Michelides Limited, in ‘Advertising’, *To Ethnico Vema*, 28 October 1931, 3. Figure 12. long description.

**Figure 13.**
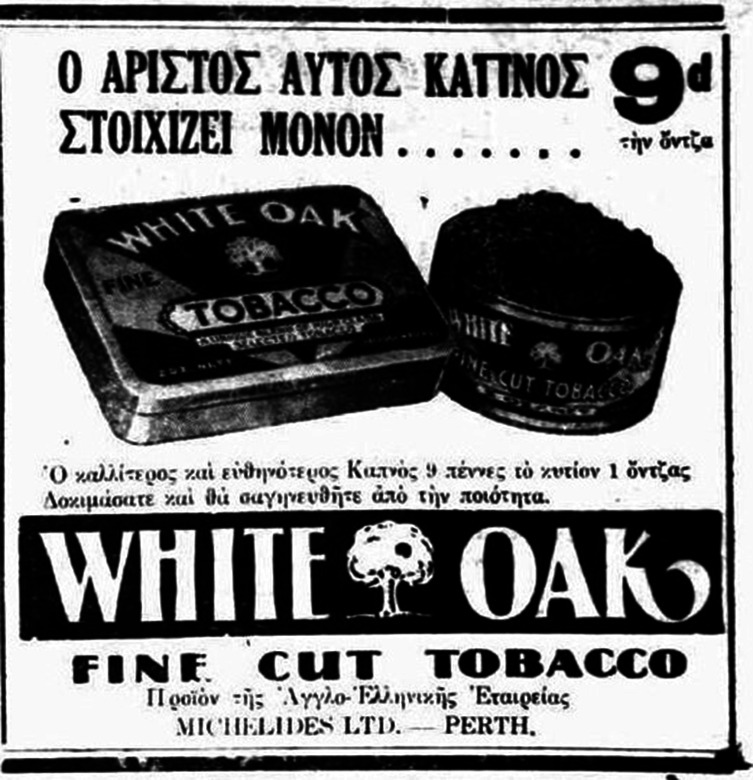
‘Advertising’, *To Ethnico Vema*, Arncliffe NSW, 15 August 1931, 6. Figure 13. long description.

**Figure 14.**
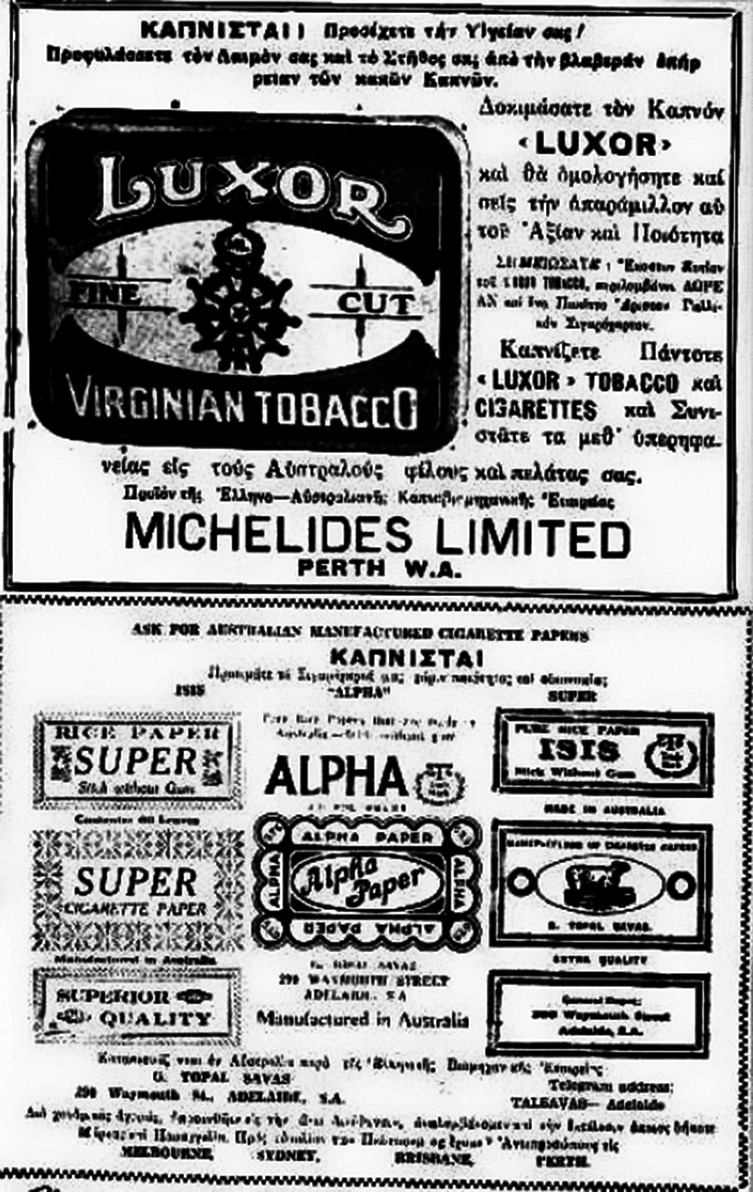
Michelides Limited and Alpha Paper advertised together in ‘Advertising’, *To Ethnico Vema*, Arncliffe NSW, 3 August 1932, 3. Figure 14. long description.

**Figure 15.**
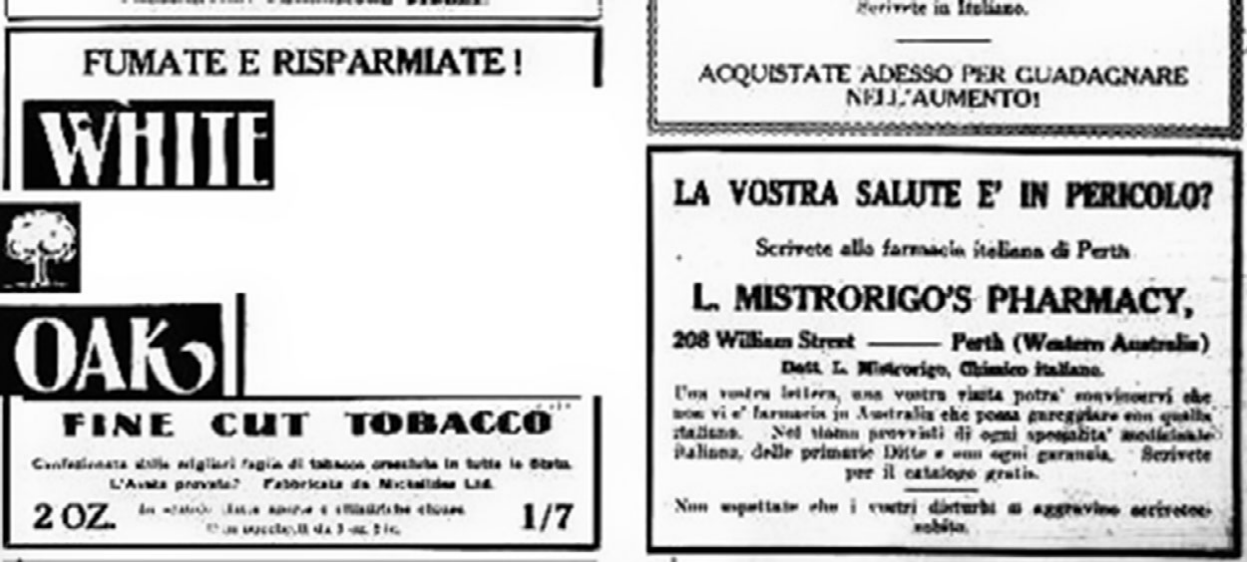
Michelides’ White Oak advertised next to the *Stampa Italiana* owner, Luigi Mistrorigo’s pharmacy, in ‘Advertising’, *Stampa Italiana*, Perth, 26 August 1932, 3. Figure 15. long description.

Michelides Limited (Figure 10), the Perth-based Greek Australian owned cigarette manufacturers, dominated advertisements in *To Ethnico Vema* (Figures 11, 12, and 13) during the 1930s. Occasionally, they were even placed alongside other Greek Australian-owned tobacco products, such as Adelaide-based Alpha Paper, owned by G. Topal Savvas (Topalsavvas) (Figure 14).^98^ Michelides Limited even expanded beyond the Greek community, being advertised in mainstream newspapers, with sensational reports, such as ‘Best Tobacco in World: Grown in Westralia…One Peter Michelides is the man who is placing Westralia on the road to be one of the greatest tobacco growing countries in the world’,^99^ and in the Italian-language newspaper *Stampa Italiana* in 1932, alongside the fascist newspaper owner, Luigi Mistrorigo’s pharmacy advertisement (Figure 15).^100^ This was reminiscent of other Greek tobacco tycoons, such as Cairo-based manufacturers like Miltiades Melachrino and Theodoro Vafiades, as discussed by Cox, who had growing dominance in British markets beginning in the 1880s and expanding into the twentieth century.^101^ Michelides’ dominance was also a talking point in *To Ethnico Vema*, with discussions of factory expansions, growing balance reports, and manufacturing hardships being commonplace.^102^ Dimitreas also noted that Michelides was a major employer of Greek migrants, notably during the 1930s, and especially due to anti-immigrant sentiments in Anglo-Celtic-dominated industries.^103^

In 1956, Michelides Limited, among other suppliers, including Italian Australian tobacco grower Tognolini, even submitted a government tender to supply plug tobacco to Aboriginal Australians in the Northern Territory.^104^ These tenders were sought due to White Australia’s assimilation policies, as it was believed ‘that as the natives advance along the road of assimilation, they become more selective in their tastes’, and that ‘The pleasures derived from the smoking or chewing of tobacco were therefore well established in the Aboriginal, and the supply of plug tobacco was only fulfilling a natural want’.^105^ This positioned Michelides as one of the government’s trusted and most utilised suppliers, until the company’s closure in 1960.^106^

In early 1950, there was a tobacco shortage, of which migrants were blamed for contributing to. There were headlines in newspapers such as ‘Migrants Mean More Smokers’ and ‘Migrants make distribution more difficult’, as paraphrased from a speech given by V. Martin, president of the Queensland Shopkeepers Association.^107^ One tobacconist noted that ‘we have had a large number of migrants, both British and European, since the war. Their smoking numbers would add considerably to the consumption figures’.^108^ Interestingly, the lack of female labour in stripping tobacco leaves was also used as a reason for the shortage, as ‘it is not economical to use male labour on this work’.^109^ However, at the same time, it was noted that there were ‘plenty of cigarettes’, and that migrant camps and enterprising new migrants were and had been the places to go to buy them both affordably and in larger quantities.^110^ Migrants were also provided with controlled tobacco cigarette rations, purchased via tobacco permits, especially for those heading to the Australian Capital Territory.^111^

This showed that tobacco control was directly connected to Australia’s internal policies, especially around migrants and minorities. Additionally, cigarette and smoking paraphernalia advertising as well as pro-cigarette smoking were significant features of migrant newspapers in Australia, including brands from their ‘old countries’, as well as of official material provided to new migrants. This was fuelled by, and continued to fuel, the tobacco and in particular cigarette culture of notably Mediterranean migrants, but also of other migrants. This was in conjunction with the ‘smoko’ culture of Australia, in which working-class migrants were particularly enveloped. However, at the same time, there was the emergence of anti-smoking messages, albeit within the framework of anti-cancer messages at first.

## Anti-cancer messages

As might be expected, until the 1950s, anti-cancer messages took precedence over any anti-cigarette messages. Discussions of emerging cancer research appeared in migrant newspapers between 1931 and 36, following the establishment of anti-cancer organisations across Australia, although much of this was intertwined within broader health advice.^112^ Many periodicals, such as the Italian-language newspapers *Stampa Italiana* (*Italian Press*) and *Il Giornale Italiano* (*Italian Newspaper*), and French-language paper *Le Courrier Australien* (*Australian Courier*), displayed an interest in cancer research and statistics, from both Australian and European perspectives.^113^ Between 1936 and 37 it appeared that Australian anti-cancer campaigns began reaching out to migrant communities, with migrant newspapers being utilised as a mode of communication. This included discussion of the 8th National Cancer Conference in Canberra in the Greek newspaper *To Ethnico Vema* (*National Tribune*), as well as statistics demonstrating rising cancer rates in NSW, becoming the second highest killer behind heart disease, and the announcement of the *Αντικαρκινικός Πόλεμος* (’Anti-Cancer War’),^114^ and an Anti-Cancer Appeal ‘card party’ organised by the Jewish community and Council of Jewish Women in Victoria, advertised in the *Australian Jewish Herald* and the *Age*, which aimed to support the Anti-Cancer Research Fund.^115^ The *Hebrew Standard of Australasia* also publicised the 2nd International Conference of the Scientific and Social Cancer Campaign, held at Brussels in 1936, which noted that ‘forty-two countries, including Palestine, participated’,^116^ as well as advertising the recipients of Cancer Research Scholarships at the Hebrew University of Jerusalem.^117^ This outreach was followed by a significant increase in cancer-related articles in migrant press, especially in *To Ethnico Vema*, *Il Giornale Italiano*, and *Le Courrier Australien*, starting from 1938, with topics ranging from international cancer campaigns, to speculations on the origins of cancers.^118^ The *Australian Jewish Herald* also followed this, recommending the Victorian Anti-Cancer Council’s publication *What Every Adult Should Know About Cancer* in 1940, aiming to educate their community and encourage ‘an increase in the early treatment of many remedial cancers’.^119^

Throughout the 1940s, the anti-cancer messages increased, especially post-WWII. In October 1941, *To Ethnico Vema* published a detailed article titled ‘Early diagnosis and treatment of cancer’, encouraging readers to watch for warning signs and act early.^120^ This was followed throughout the decade with other awareness pieces, which urged for diagnosis, and discussed treatments and statistics, with prostate, breast, skin, and gastro-intestinal cancers often being of focus.^121^ In 1946, after the end of the Second World War, there appeared to be significant cross-communication between the *Sydney Jewish News* and the Anti-Cancer Council Victoria, where it was noted at length that:

The Council desires to stress to the public of its State its conviction of the paramount importance of early treatment in the cure of cancer, and of the urgent necessity which exists, therefore, that patients suffering from this disease should report early for advice to their local medical practitioner or nearest public hospital.^122^

The Anti-Cancer Council’s free handbook was again advertised in 1946, and messages encouraging early treatment spilled over into the *Australian Jewish Herald.* ^123^ Hopeful messages for successful treatments, especially using radium for skin, lip, and breast cancers, and hormones for breast cancer, were widely published in migrant newspapers.^124^ In 1946 *Le Courrier Australien* published a four-paragraph English-language article on the British Empire Cancer Campaign, noting its fundraising and research strengths.^125^ This was alongside an almost full-page piece on French cancer research, which discussed X-ray, hormone, and radium therapies, and emphasised that due to the boom in research centres, ‘France must play an important part in the “final victory”’.^126^ Foreign anti-cancer campaigns also made their way into migrant newspapers, which additionally highlighted the need for early diagnosis as a key to fighting tumours. These campaigns included the Italian League for the Fight Against Cancer, discussed in the Sydney-based *Il Risveglio*, and the Anti-Cancer Campaign of Athens, which commenced in 1946 and was discussed in *To Ethnico Vema* that year.^127^ There were also frequent claims of ‘cures’ published in the 1940s, such as Italian Dr Guarnieri who used ‘a harmonic drug called FA2’ to cure cancer.^128^ Despite the post-war context, the anti-cancer messages and other media ultimately still seemed rooted in the 1930s.

During the 1950s, there was an increased awareness of the seriousness of cancer and the importance of acting on it in migrant press. This importance translated into discussions of hope and future cures. In the early 1950s, there was a boom of discussion around new cancer treatments, with research from the US and Europe finding its way into papers like *To Ethnico Vema.* Two 1951 articles discussed the hopefulness of cancer researchers in Washington and Greece who believed a cure was near, with research from Johns Hopkins University being published, which described chemical compounds that starved and killed cancer cells.^129^ While another article, from January 1952, saw atomic energy and radioactive liquid gold used by American scientists as a possible solution to prostate and cervical cancers, and leukaemia.^130^ Annual $10,000 grants for cancer research at the Hebrew University Hadassah Medical School, donated by the Damon Runyon Memorial Fund, were noted widely between 1951 and 53.^131^ Other theories were put forward in early 1952 including American doctors linking cancer and thrombosis; Viennese Prof Leopold Senbauer who believed cancer to be hereditary; Italian Dr Clara Jolles-Fonti who experimented with a cancer vaccine on herself and claimed cancer was contagious; and Greek Prof Aristotelis P. Kouzis who looked at X-ray and nitrogen mustard treatments.^132^ These hopeful messages for cures were not only derived from international research papers – in July 1952 *To Ethnico Vema* published an article quoting Dr A.B. Lilley, president of the NSW Hospital Board, who had liaised with Australian philanthropist Sir Edward Hallstrom after his visit to the American Cancer Institute, stating ‘that scientists will very soon find the answer to the disease of cancer, where a huge advance in cancer research gave great hope’.^133^

Into the 1950s, other creative ways of fighting cancer were presented to Greek readers. In February 1953, Britain was using atomic isotopes to cure leukaemia,^134^ while in 1954 Dr Eugene Payne was examining South American natives to find what caused their resistance to cancer and other diseases,^135^ and a British filmmaker had been cured due to his religious faith.^136^ Jolles-Fonti’s research would feature again, this time in Italian-language newspapers, such as Sydney’s *Il Risveglio*, in 1955, where it was noted that she had presented theories on cancer originating from an endocrine imbalance or from a parasite.^137^ In 1958, the *Australian Jewish News* continued Australia’s Jewish communities’ wide support for anti-cancer campaigns, when it was noted that a ‘Mr. Stanley Korman, well-known member of the Melbourne Jewish Community, who is equally well-known in Queensland for his financial activities in Surfers Paradise, recently presented a personal cheque for £2,500 for the Anti-Cancer Campaign’,^138^ and that a multi-team soccer premiership would be played at Olympic Park, Melbourne, in order to fundraise for the Anti-Cancer Campaign (whether this was the state or federal campaign is unclear).^139^

These discussions, although often hopeful and positive, were not always useful in providing key preventative information for migrant readers in Australia, or even providing clear avenues for prospective donations or support. Then, during the 1950s, anti-smoking discussions began, which saw greater awareness of tobacco’s dangers and the beginning of preventative measures for lung cancer. These discussions were juxtaposed with the contradictory and frequent tobacco product advertisements. These discussions were the basis for the shift that led to eventual tobacco advertisement bans, tobacco sale restrictions, and cancer-risk awareness laws, which have been ongoing since the 1970s, with the first Federal education campaign being the ‘National Warning Against Smoking’ between 1972 and 75, and the implementation of health warning legislation and cigarette broadcast bans in 1973.^140^

## Anti-smoking messages

Within the growing push for anti-cancer information to become widespread in Australia and among migrants, there was a simultaneous discussion around smoking, coupled with anti-smoking messages. These messages, however, were competing with the aforementioned tobacco advertising and corporate and cultural denialism, and did not take great hold until the 1970s, as scholars have acknowledged.^141^ As early as the 1920s and 1930s, there was some understanding of tobacco and its links to cancer (presumably lung) in Australia. In 1928 Robert Imray Esq. of Greymare, Queensland (a notable sawmill owner), petitioned Prime Minister Bruce, believing there was a link between newly manufactured tobacco products (especially cigarettes) and cancer.^142^

By the 1930s, these types of discussions were entering migrant newspapers, which published on the dangers of smoking and tobacco and its links to mouth and lip cancer. This was evident in 1933 when both *Il Giornale Italiano* claimed that tobacco ‘causes that most fearful and terrible cancer’, and *To Ethnico Vema* discussed French pathologist Charles Imbert’s attribution of mouth, lip, and larynx cancer to smoking.^143^ Smoking in public places did cause concern among some members of migrant communities pre-1950s, with the NSW Theatres and Public Halls Act (1908) indirectly prohibiting smoking in theatres and public halls without a license, particularly for the sake of fire safety, air ventilation, and the public health risks resulting from these factors.^144^ This was seen emphasised in a 1946 ‘Letter to the Editor’ in the *Hebrew Standard of Australasia*, which complained that:

Sir- At a recent public meeting at the Macabean Hall, at which a Federal member was present, it was observed that a number of the public present was smoking. Discomfort caused to the objectors need hardly be stressed. No doubt some visitors and strangers are unacquainted with the local law, which prevents smoking in a public hall. Offenders are asked to avoid repetition of similar occurrences. Yours, etc., J.P.^145^

After 1947, *Il Risveglio*, in true anti-capitalist fashion, published a piece arguing that nicotine was bad for the heart and ‘is a poison that ruins many smokers. On the other hand, capitalists make money by the barrel’.^146^ The article also discussed Dr Harold J. Stewart, who was a professor of clinical medicine at Cornell University, New York, and his research which demonstrated that ‘once again that tobacco is the enemy of the heart. Sixteen men aged 21 to 38 took the test. The experiment proved beyond dispute that tobacco damages the arteries and therefore the heart’.^147^ These types of research articles displayed a growing discourse and acknowledgement around the dangers of smoking within migrant communities, even before the 1950s.

Anti-smoking messages appeared to increase significantly in the Australian migrant and minority papers explored during the 1950s. This followed the link between tobacco and lung cancer being confirmed in scientific research. These messages discussed and debated findings around scientific discoveries and their validity. 1952 appeared to have an influx of these types of articles in *To Ethnico Vema*, with headlines including ‘Smoking and Cancer’, which discussed the research of Dr Max Torek linking smoking and cancer;^148^ and the large article titled ‘Lung cancer is caused by asphalt fumes and cigarettes!’, which discussed growing sentiments in Europe around these causes.^149^ Other articles featured throughout the decade provided a motley of different viewpoints, questions, and solutions, such as Swiss physician Dr Steiner who manufactured the anti-cancer cigarette that he himself smoked regularly,^150^ as well as the argument that smoking was dangerous mostly for those who were already suffering from other illnesses.^151^

There were some smoking apologists who made headlines in *To Ethnico Vema*, such as the aptly named ‘In defence of cigarettes, drinks, and coffee’.^152^ This article ultimately presented the anti-cigarette messages as an attack on cultural norms rather than being health related. Others at least questioned the current research, such as the 1953 semi-sceptical article titled ‘Is smoking a causative agent of lung cancer?’, which drew on the research of British Professor Richard D. Passey of the University of Leeds, who argued that there needed to be a ‘a systematic examination a group of smokers in parallel with a group of non-smokers’ before conclusions could be drawn.^153^ Passey himself was rebutted by Doll, Hall, Binysh, and Johnston that same year in the *British Medical Journal.* ^154^ Passey further would claim, along with Professor F.W. Spiers, that ‘Such radioactivity as is present in cigarette tobacco remains in the ash and very little, if any, is transferred in the smoke’.^155^

However, by 1954 and beyond, there was less scientific doubt, and stronger statistics being presented to migrant readers. One article titled ‘Lung cancer’ noted that lung cancer in men had risen by 500% since 1934 according to the American Cancer Society, and that smoking was considered the ‘number 1 risk’, although it did add that some scientists ‘are not entirely convinced’.^156^ A similar article reported that ‘Lung cancer in Great Britain is constantly gaining ground: 5 1/2 times more men than women die. The victims of all types of cancer during the past year reached 87,924’.^157^ However, tobacco or smoking was not mentioned here. An extended 1957 article in *Elefthera Phoni* on ‘The English, smoking and lung cancer’, however, dispersed any doubt about the link, and went into detail about a House of Commons debate which stated that ‘It is now well known that smokers have a higher mortality rate from lung cancer’.^158^ It also went on to discuss the research of Sir Henry Cohen which noted that non-smokers had ‘a minimal 0.5% chance of developing cancer, whereas a smoker has an 8% chance’.^159^ *Elefthera Phoni* published a similar article half a month later noting that American statisticians found that ‘cancer of the mouth, throat and lungs is more frequent among smokers than among non-smokers’.^160^

By 1957, the *Australian Jewish News* clearly and consistently confirmed the link between smoking and cancer:

A connection between smoking and lung cancer had definitely been established and heavy smokers were doing so at their own peril, according to Dr. Ronald Rosanove, prominent Melbourne medico and scientist, now here on a visit from the U.S.A…Dr. Rosanove…said that Australians had the tendency to regard everything American as good. This was not necessarily so, and, although Australians could learn a lot from the United States, it was essential that she remain independent and maintain close ties with the United Kingdom and the British Commonwealth.^161^

Furthermore, the paper acknowledged in 1956 the ‘Power of the Press’ in convincing community members to give up smoking, while simultaneously urging and encouraging other readers to do the same:

**Power of the Press**

‘And then there is the highly amused wife of one of our best-known communal workers who is one of the finest cancer experts in this State…

It seems that said expert has given up smoking. Explains his loving wife that he read an article on “Smoking and Cancer” in “The Reader’s Digest” and got such a fright he hasn’t had a cigarette since’.^162^

These changes around 1957 coincided with the Australian National Health and Medical Research Council (NHMRC) first raising the issue of smoking with the Minister of Health, and called for anti-smoking campaigns in all states. This encouraged undeniable discussions in migrant newspapers. Left-wing Greek newspaper *Neos Kosmos* (*New World*), which launched in February 1957, took this on board, with articles such as ‘The Cancer-Smoking Relationship is Undoubted’ featuring within its 2 months.^163^ These messages only increased into the early 1960s.^164^

These examples of anti-cancer messages in migrant newspapers were aimed at the readership of migrant communities during the 1950s. They hint at a care and sense of responsibility evident from the newspaper editors towards their communities in reporting anti-smoking developments. They speak to the important role played by the migrant and minority press in the lives of diaspora members, yet there does not appear to be a great deal of coordination or guidance on the subject. This may be a reflection on the developing approach to anti-smoking everywhere, or perhaps on the Australian case. The references to international, especially European, information and developments in anti-smoking indicated a preference to look to the ‘old world’ of Europe for leadership and guidance, perhaps reflecting the lack of leadership and guidance in Australia during the 1950s on this subject.

## Postscript and conclusion

On 17 May 1960 the federal Minister for Health, Donald Cameron, issued a statement on smoking and lung cancer in response to the findings released by the NHMRC that linked cigarette smoking to cancer. After seeking advice from the Cabinet on what to say publicly, Cameron announced that he would release the findings and make it clear that they ‘were arrived at by the highest medical authorities’, but ‘beyond that, the matter was one for private decision’.^165^

Three years later a similar decision was taken, the main difference being that the Cabinet pushed the issue back onto the states to do something if they wanted. Within those 3 years much had transpired to further confirm the link between cigarette smoking and cancer.^166^ In 1962 the Royal College of Physicians (RCP) in the UK released an expert committee review into smoking and health, which concluded that cigarette smoking was a cause of lung cancer.^167^ In response to this and its own further research, the NHMRC issued a series of recommendations to the federal and state governments to discourage smoking and to establish a research body to inquire into and recommend measures to reduce the risk to cigarette smokers of contracting lung cancer. The Attorney-General, Garfield Barwick, took these recommendations seriously and advised the federal government on what actions could be taken, such as restricting cigarette advertising through an amendment to the Broadcasting and Television Act, creating such a research body as suggested, and banning smoking in the territories and in other places as recommended by the NHMRC, amongst other suggested practical changes.^168^

Meanwhile, C.F. Aderman, the Minister for Primary Industry, prepared a memorandum on the likely impact that the recommendations of the NHMRC would have on tobacco growing and manufacturing. As might be expected, he argued that any decrease in cigarette smoking ‘would be detrimental to the Australia leaf industry’ and there would be backlash from the approximately 4,500 growers, especially in Northern Queensland, and from the US, which was the primary supplier of tobacco leaf. He further argued that any reduction in smoking would adversely impact the 5,000 workers in the industry and pointed out that tobacco was ‘a very large revenue earner’, with customs duties on the import of tobacco, cigars, snuff, and cigarettes amounting to almost £12m and revenue from excise totalling almost £80m.^169^

The Minister for Health, H.W. Wade, presented a detailed report starting with the evidence and statistics supporting the evidence of a link between cigarette smoking and lung cancer. He showed that since 1950 the death rate from lung cancer in males had doubled from 150 per million to 306 per million, mostly in males over 55, and that the number of deaths due to lung cancer had almost tripled in 10 years. He referred to international studies, and specifically to the RCP report, to argue that ‘there is no longer any doubt that the increasing incidence of lung cancer is real, easily demonstrable, and is not related primarily to improvements in diagnosis and treatment’. He then summarised the various recommendations of the NHMRC from May 1957 and then the latest ones, which included measures that both the Commonwealth and state governments could take to decrease cigarette smoking. He ended by asking for the direction of the Cabinet on whether he should endorse and implement the recommendations of the NHMRC.^170^ The Cabinet had a robust discussion on the subject on 1 February 1963, acknowledging the problem and that action was needed, but did not want Wade to move ‘too fast’, wanting more evidence and to not upset or anger the industry or the newspapers and broadcasting and television stations. It also wondered about increasing excise duties to offset the reduction in smoking, and perhaps ask the treasury and customs on this point.^171^ Ultimately, 4 days later the Cabinet decided to do nothing other than to push the matter back onto the states.^172^

This laissez-faire and handballing to the states reflected the inconsistent and confused messages in the migrant newspapers during the 1950s. Migrants to Australia, especially from Southeastern Europe and the Mediterranean, came from countries with established tobacco-growing and cigarette manufacturing industries and with high rates of cigarette smoking. This made them sought after in Australia for their knowledge as well as for their labour. Many migrants, especially from Italy, Greece, Yugoslavia, Malta, and Cyprus, owned tobacco-growing farms and employed people from their countries and migrants in general. It was not in their interests to embrace anti-smoking messages. Their relationship to cigarette smoking from before their emigration also made them more susceptible to advertising, which was prevalent both before 1950 and after, and to the Australian ‘smoko’, which they seemed to embrace. It also made them more resistant to anti-cancer messages when the link to cigarettes and cancer was made, as they had a long association with cigarette smoking from before they arrived in Australia. While the newspapers reported on the research and debates, they were not generally providing much detail or taking a strong position against the dangers of cigarettes, while continuing to provide space for cigarette advertising. There was also much confusion created by the reporting of the denial of the medical evidence by the cigarette industry, causing readers to second-guess that evidence, especially since the government refused to act or even to take a strong position. This inconsistency perhaps reflected wider Australian trends and the mixed messages emanating from the government. This may have led to migrant newspaper editors seeking guidance on the matter from the international, especially European, press. From the late 1950s, there was a somewhat clearer direction on anti-smoking messages in the migrant newspapers, reflecting both international and Australian developments.

Exploring the same subject and themes for the period from 1960 would likely show similar tensions, but with the tide turning against the cigarette once the Australian government began to take meaningful action against cigarette smoking in line with international developments. This would be coupled with the various health studies on Southeastern European and Mediterranean populations in Australia in the 1970s and 1980s. It remains to be determined what the migrant press reported on the debates around curtailing cigarette smoking and the health studies relating to their migrant communities in such a future study.

## Footnotes

1
*The Australian Women’s Weekly*, 21 November 1951, 29.

2 Andrekos Varnava, ‘The Origins of “Big Tobacco” Cigarette Manufacturing and the Prevalence of Smoking in Colonial Cyprus, 1920–1960’, *Enterprise & Society*, 27, 1 (2026), 216–253. Thomas J. Kehoe , Andrekos Varnava and Elizabeth Greenhalgh , ‘British Imperial Legacies and Tobacco Power: Interrogating Connections Between Colonial Histories and Corporate Influence Over Modern Tobacco Control Measures’, Social History of Medicine, 38, 1 (2025), 37–59 .

3

Richard Doll and A. Bradford Hill , ‘Smoking and Carcinoma of the Lung’, British Medical Journal, 2, 4682 (1950), 739–4814772469 10.1136/bmj.2.4682.739PMC2038856.

4 See: Mark Clement , Smoking and Cancer: Facts That Every Smoker Ought to Know! (Sussex: Health Sciences Press, 1953).

5

E.C. Hammond and D. Horn , ‘The Relationship Between Human Smoking Habits and Death Rates: A Follow-Up Study of 187,766 Men’, Journal of the American Medical Association, 155, 15 (1954), 1316–2813174399 10.1001/jama.1954.03690330020006.

6

David T. Cartwright , ‘“Carry on Smoking”: Public Relations and Advertising Strategies of American and British Tobacco Companies since 1950’, Business History, 47, 3 (2005), 421–33.

7

Colin Talley , Howard I. Kushner and Claire E. Strerk , ‘Lung Cancer, Chronic Disease Epidemiology, and Medicine, 1948–1964’, Journal of the History of Medicine and Allied Sciences, 59, 3 (2004), 329–7415270334 10.1093/jhmas/jrh088.

8

Robert N. Proctor , ‘The History of the Discovery of the Cigarette-Lung Cancer Link: Evidentiary Traditions, Corporate Denial, Global Toll’, Tobacco Control, 21, 2 (2012), 87–91 22345227 10.1136/tobaccocontrol-2011-050338.

9

Matthew Hilton , *Smoking in British Popular Culture, 1800–2000*, Studies in Popular Culture (Manchester & New York: Manchester University Press, 2000), 241 .

10 Royal College of Physicians of London, *Smoking and Health: A Report of the Royal College of Physicians on Smoking in Relation to Cancer of the Lung and Other Diseases* (London: Pitman, 1962). See also Virginia Berridge , ‘The Policy Response to the Smoking and Lung Cancer Connection in the 1950s and 1960s’, The Historical Journal, 49, 4 (2006), 1185–20920200590 10.1017/S0018246X06005784PMC2829889.

11

Ian Tyrrell , Deadly Enemies: Tobacco and Its Opponents in Australia (Sydney: UNSW Press, 1999), 193–5.

12 A. Toth-Peter, R.G. Richards, S. Reid, C. Gartner, S. Staby and C. Smith, ‘Smoking Model: Evaluating the Dynamics of Tobacco Smoking in Australia’, 3rd Asia-Pacific System Dynamics Conference, University of Queensland, Brisbane, 3-4 February 2020, 1.

13 Australian Government, Department of Health and Aged Care, ‘Tobacco Control Timeline’, 10 May 2018, accessed 19.02.2024: https://www1.health.gov.au/internet/publications/publishing.nsf/Content/tobacco-control-toc~timeline.

14

Iain Gately , Tobacco: A Cultural History of How an Exotic Plant Seduced Civilization (New York: Grove Atlantis, 2007).

15

Howard Cox , The Global Cigarette: Origins and Evolution of British American Tobacco, 1880–1945 (Oxford & New York: Oxford University Press, 2000), 333–47.

16 Hilton, *op. cit.* (note 9), 241.

17 Tyrrell, *op. cit.* (note 11), xi–xii. See also: Ian Tyrrell , ‘Before the Surgeon-General’s Report: Public Discourse in Australia Over Tobacco Addiction to 1964’, Australian Journal of Politics & History, 44, 2 (1998), 177–90; Ian Tyrrell , ‘The Anti-Tobacco Reform and the Temperance Movement in Australia: Connections and Differences’, Journal of the Royal Australian Historical Society, 84, 1 (1998), 10–25 .

18

Steve Engleman , ‘The Impact of Mass Media Anti-Smoking Publicity’, Health Promotion International, 2, 1 (1987), 63–74; Warren Breed and James R. De Foe , ‘Drinking and Smoking on Television, 1950–1982,’ Journal of Public Health Policy, 5, 2 (1984), 257–706470134 .

19

Evan Smith , Andrekos Varnava and Marinella Marmo , ‘The Interconnectedness of British and Australian Immigration Controls in the 20th Century’, International History Review, 43, 6 (2021), 1354–74; Eric Richards , Destination Australia: Migration to Australia Since 1901 (Manchester & Sydney: Manchester Uni Press and UNSW Press, 2008); James Jupp , Immigration (Melbourne: Oxford University Press, 1998); James Jupp , Arrivals and Departures (Melbourne: Cheshire-Lansdowne, 1966).

20

Andrekos Varnava , ‘Understanding the Support From the Australian Far-Left and ALP-Left for Greek Cypriot Enosis During the EOKA Period (1955–59): Migrant Workers, Anti-Imperialism and National Liberation in Australia’, Labor History, 64, 2 (2023), 165–84; Andrekos Varnava , ‘Border Control and Monitoring “Undesirable” Cypriots in the UK and Australia, 1945–1959’, Immigrants and Minorities, 40, 1–2 (2022), 132–76; Evan Smith , Andrekos Varnava and Marinella Marmo , ‘The Interconnectedness of British and Australian Immigration Controls in the 20th Century’, International History Review, 43, 6 (2021), 1354–74; Yianni Cartledge and Andrekos Varnava , ‘Making and Monitoring a “Suspect Community”: Australian Attacks on Greeks and the “Secret Census” in 1916’, Australian Historical Studies, 55, 3 (2024), 485–505 ; Yianni Cartledge, ‘Beyond the “Red Rock”: The Ikarian Revolution (1912), Political Radicalism, and the Ikarian Diaspora’, *Labor History*, 66, 5 (2024), 659–77; Yianni Cartledge , Ikarians in South Australia, 1900–1945: Emigration, Settlement, Community Building and Integration (London: Anthem Press, 2026).

21 Catherine Dewhirst and Richard Scully, *The Transnational Voices of Australia’s Migrant and Minority Press* (London: Palgrave, 2020); Catherine Dewhirst and Richard Scully, *Voices of Challenge in Australia’s Migrant and Minority Pres*s (London: Palgrave, 2021).

22 Alison Culpin, Suzanne Gleeson, Margaret M. Thomas, and Jenny Bekiaris, ‘Evaluation of the “Good Heart, Good Life” (*Καλή Καρδιά, Καλή Ζωή*) Project: A Three-Year Campaign to Reduce Smoking Among the Greek Community of Sydney’s Inner West’, *Health Promotion Journal of Australia*, 6, 2 (1996), 44–9.

23 See: George Donatos , ‘Advertising, Anti-Smoking Campaign, and Cigarette Consumption in Greece’, European Research Studies, VII, 3–4 (2004), 1–13 ; Vasilios G. Stavrinos , ‘The Effects of an Anti-Smoking Campaign on Cigarette Consumption: Empirical Evidence From Greece’, Applies Economics, 19, 3 (1987), 323–9.

24

Lisa Trotter , ‘Survey of Smoking Beliefs and Behaviours in Greek and Chinese Communities’, Quit Evaluation Studies, 9 (1996–97), 107–20.

25

Masoud Mohammadnezhad , George Tsourtos , Carlene Wilson , Julie Ratcliffe , and Paul Ward , ‘Understanding Socio-Cultural Influences on Smoking Among Older Greek-Australian Smokers Aged 50 and Over: Facilitators or Barriers? A Qualitative Study’, International Journal of Environmental Research & Public Health 12, 3 (2015), 2718–3425739006 10.3390/ijerph120302718PMC4377928, at 2730–1. See also: Masoud Mohammadnezhad , George Tsourtos , Carlene Wilson , Julie Ratcliffe , and Paul Ward , ‘“I Have Never Experienced Any Problem With My Health. So Far, It Hasn’t Been Harmful”: Older Greek-Australian Smokers’ Views on Smoking: A Qualitative Study’, BMC Public Health, 15, 304 (2015), 1–11 25885899 10.1186/s12889-015-1677-6PMC4391085.

26

Myriam Khlat , ‘Cancer in Mediterranean Migrants – Based on Studies in France and Australia’, Cancer Causes & Control, 6 (1995), 525–318580301 10.1007/BF00054161.

27 See: Peter Bardsley and Nilss Olekalns , ‘Cigarette and Tobacco Consumption: Have Anti-Smoking Policies Made a Difference?’, *The Economic Record*, The Economic Society of Australia, 75, 3 (1999), 225–40; David T. Courtwright , “Carry on Smoking’: Public Relations and Advertising Strategies of American and British Tobacco Companies Since 1950’, Business History, 47, 3 (2005), 421–33; Virginia Berridge and Kelly Loughlin , ‘Smoking and the New Health Education in Britain 1950s–1970s’, American Journal of Public Health, 95, 6 (2005), 956–6415914816 10.2105/AJPH.2004.037887PMC1449291; John P. Pierce , ‘Australia’s Leadership Role in the Evolution of Tobacco Control Programs in the United States’, Health Promotion Journal of Australia, 7, 1 (1997), 11–7; Claire Hooker and Simon Chapman , ‘Structural Elements in Achieving Legislative Tobacco Control in NSW, 1955–95: Political Reflections and Implications’, Australian and New Zealand Journal of Public Health, 30, 1 (2006), 10–516502946 10.1111/j.1467-842x.2006.tb00078.x.

28

David Cantor , ‘Introduction: Cancer Control and Prevention in the Twentieth Century’, Bulletin of the History of Medicine, 81, 1 (2007), 1–38 10.1353/bhm.2007.000117369661; Stephen Snelders , Frans J Meijman and Toine Pieters , ‘Cancer Health Communication in the Netherlands 1910–1950: Paternalistic Control or Popularization of Knowledge?’, Medizinhistorisches Journal, 41, 3–4 (2006), 271–8917265849 ; Elizabeth Toon , ‘“Cancer as the General Population Knows It”: Knowledge, Fear, and Lay Education in 1950s Britain’, Bulletin of the History of Medicine, 81 (2007), 116–3817369665 10.1353/bhm.2007.0013PMC2635844. See also: Ornella Moscucci , ‘The British Fight Against Cancer: Publicity and Education, 1900–1948’, Social History of Medicine, 23, 2 (2009), 356–73; Hannah J. Elizabeth , Gareth Millward and Alex Mold , “Injections-While-You-Dance’: Press Advertisement and Poster Promotion of the Polio Vaccine to British Publics, 1956–1962’, The Journal of the Social History Society, 16, 3 (2019), 315–3632104153 10.1080/14780038.2019.1586061PMC6999345.

29 See: Toth-Peter et al., *op. cit.* (note 12), 1–10; David Reubi and Virginia Berridge , ‘The Internationalisation of Tobacco Control, 1950–2010’. Medical History, Cambridge University Press, 60, 4 (2016), 453–7227628857 10.1017/mdh.2016.97PMC5058405; Rosemary Elliot , ‘Inhaling Democracy: Cigarette Advertising and Health Education in Post-War West Germany, 1950s–1975’, Social History of Medicine, 28, 3 (August 2015), 509–3126217071 10.1093/shm/hkv004PMC4513888.

30

Kenneth E. Warner , ‘The Effects of the Anti-Smoking Campaign on Cigarette Consumption’, American Journal of Public Health, 67, 7 (1977), 645–50879393 10.2105/ajph.67.7.645PMC1653696; Kenneth E. Warner , ‘Effects of the Antismoking Campaign: An Update’, American Journal of Public Health, 79, 2 (1989), 144–512913831 10.2105/ajph.79.2.144PMC1349923; John P. Pierce , Petra Macaskill and David Hill , ‘Long-Term Effectiveness of Mass Media Led Antismoking Campaigns in Australia’, American Journal of Public Health, 80, 5 (1990), 565–92327533 10.2105/ajph.80.5.565PMC1404648.

31

Robert N. Proctor , Golden Holocaust: Origins of the Cigarette Catastrophe and the Case for Abolition (Berkeley, Los Angeles & London: University of California Press, 2011); Sarah Milov , The Cigarette: A Political History (Cambridge, MA: Harvard University Press, 2019); Philip DeCicca and Logan McLeod , ‘Smoking in Canada’, in Dean Lilla and Rebekka Christopoulou (eds.), Life-Course Smoking Behavior: Patterns and National Context in Ten Countries (Oxford: Oxford University Press, 2015), 29–42 ; Cook, Sharon Anne , Sex, Lies and Cigarettes: Canadian Women, Smoking and Visual Culture, 1880–2000 (Montreal: McGill-Queen’s University Press, 2012); Mark Asbridge , ‘Public Place Restrictions on Smoking in Canada: Assessing the Role of the State, Media, Science and Public Health Advocacy’, Social Science & Medicine, 58, 1 (2004), 13–24 14572918 10.1016/s0277-9536(03)00154-0; Rob Cunningham , Smoke & Mirrors: The Canadian Tobacco War (Ottawa: International Development Research Centre, 1996); Robin Berwick Walker , Under Fire: A History of Tobacco Smoking in Australia (Melbourne: Melbourne University Press, 1984); Tyrrell, *op. cit.* (note 11).

32 Maltese language newspapers are a notable omission, due to the significant Maltese presence in Australia. This is due to the first Maltese language paper, the *Maltese Herald*, launching in 1961.

33 See: Dewhirst and Scully (eds.), *op. cit.* (note 21); Dewhirst and Scully (eds.), *op. cit.* (note 21).

34 See: Marianna Piantavigna, ‘“Cement, Guide and Representative for the Exile and the Emigrant”: Ideological Discourse and *italianità* in *L’Italo-Australiano*’, in Dewhirst and Richard Scully (eds.), *The Transnational Voices of Australia’s Migrant and Minority Press*, 37–59; Catherine Dewhirst, ‘Respectability and Disloyalty: The Competing Obligations of *L’Italiano’s* Editors’, in Dewhirst and Scully (eds.), *The Transnational Voices of Australia’s Migrant and Minority Press*, 81–105; Angela A. Alessi, ‘Reimagining Italian Spaces: *La Fiamma* as a Lens to Explore the Development of the Italian Community in Adelaide, South Australia, Between 1947 and 1963’, in Dewhirst and Scully (eds.), *Voices of Challenge in Australia’s Migrant and Minority Press*, 107–25; Clare Johansson and Simone Battiston, ‘News Reporting of Italian Organised Crime in Australia: Examining *Il Globo’s* Editorial Commentary’, in Dewhirst and Scully (eds.), *The Transnational Voices of Australia’s Migrant and Minority Press*, 189–208; Bruno Mascitelli, ‘Reflections and Transition of Old and New Italian Media in Australia: The Case of *Il Globo*’, in Dewhirst and Scully (eds.), *Voices of Challenge in Australia’s Migrant and Minority Press*, 127–45; Simone Battiston, ‘Historicising the Early Years of *Nuovo Paese* (1874–1981)’, in Dewhirst and Scully (eds.), *Voices of Challenge in Australia’s Migrant and Minority Press*, 147–71; Michael Jacklin, ‘*Crónicas* in Australia’s Spanish-Language Press: The Case of *El Expreso*’, in Dewhirst and Scully (eds.), *The Transnational Voices of Australia’s Migrant and Minority Press*, 169–87; Max Kaiser, ‘Zionism, Assimilationism and Antifascism: Divergent International Jewish Pathways in Three Post-War Australian Jewish Magazines’, in Dewhirst and Scully (eds.), *The Transnational Voices of Australia’s Migrant and Minority Press*, 107–25; Alexis Bergantz, ‘Remembering *Australasie*: European Settler and Trans-imperial Thinking in the Cosmopolitan *Le Courrier Australien* (1892–1896), in Dewhirst and Scully (eds.), *Voices of Challenge in Australia’s Migrant and Minority Press*, 43–61.

35 Migrants from Egypt primarily had Maltese, Greek and Cypriot heritage, and were fleeing Colonel Nasser’s Arabisation program.

36 Suzanne D. Rutland, ‘Post-War Jewish Migration’, *Israel & Judaism Studies*, 2018: https://ijs.org.au/post-war-jewish-migration.

37

George Kanarakis , ‘The Press of Greeks in Australia: With Reference to Other Presses of the Hellenic Diaspora’, Journal of the Hellenic Diaspora, 18, 2 (1992), 111–27.

38

Miriam Gilson and Jerzy Zubrzycki , The Foreign-language Press in Australia, 1848–1964 (Canberra: ANU Press, 1967), 19 & ix; George Kanarakis , ‘A Page From the History of the Greek Presence in the Pacific Rim’, in E. Close , M. Tsianikas and G. Frazis (eds.), Greek Research in Australia: Proceeding of the Biennial International Conference of Greek Studies, Flinders University April 2003 (Adelaide: Flinders University, 2005), 215–30, at 317.

39 Census of the Commonwealth of Australia, 1933: Part X Birthplace, 728–30; Part XVI Religion, 1020–78. http://www.abs.gov.au/AUSSTATS/abs@.nsf/DetailsPage/2110.01933?OpenDocument; Census of the Commonwealth of Australia, 1947: Part XII Birthplace, 638–40; Part XVI Religion, 872–927. http://www.abs.gov.au/AUSSTATS/abs@.nsf/DetailsPage/2109.01947?OpenDocument; Census of the Commonwealth of Australia, 1954: Volume VIII, Part I, Cross-classifications of the characteristics of the population, 22 & 64–5. http://www.abs.gov.au/AUSSTATS/abs@.nsf/DetailsPage/2109.01947?OpenDocument; Census of the Commonwealth of Australia, 1961: Volume VIII, Part I, Cross-classifications of the characteristics of the population, 27 & 78–9. http://www.abs.gov.au/AUSSTATS/abs@.nsf/DetailsPage/2107.01961?OpenDocument; Census of Population and Housing.

40 Gilson and Zubrzycki, *op. cit.* (note 38), 32; Kanarakis, *op. cit.* (note 38), 317; Christos N. Fifis , ‘The Pre-World War II Greek Community of Australia: Class Divisions and Trends’, Journal of the Hellenic Diaspora, 30, 2 (2004), 57–83, at 78; Toula Nicolacopoulos and George Vassilacopoulos , ‘The Making of Greek-Australian Citizenship: From Heteronomous to Autonomous Political Communities’, Modern Greek Studies (Australia & New Zealand), 11 (2003), 165–76, at 170–1.

41 ‘Η Αρχή (The Beginning)’, *Elefthera Phoni*, Perth, 20 July 1956, 1. See: John N. Yiannakis, “Get yer ya yas Out’: Aspects of Perth’s Greek Australian Youth Culture From the 1950s to the 1970s’, *Modern Greek Studies (Australia & New Zealand)*, special issue: Thinking Diversely, 2012, 290–1.

42 Gilson and Zubrzycki, *op. cit.* (note 38), 19.

43 Bergantz, *op. cit.* (note 34), 43–4.

44 Jean Rosemberg, *Studies in the French Presence in Australia*, unpublished typescript, Melbourne, 1987, 110–1.

45 Suzanne D. Rutland, ‘Alfred Harris (1870–1944)’, *ADB*, 9 (1983).

46 See: Shmuel Bennett, *Chronicles of a Life*, S. Bennett, Melbourne, 1999; Suzanne D. Rutland , Pages of History, A Century of Australian Jewish Press (Melbourne: Australian Jewish Press, 1995).

47 Suzanne D. Rutland, ‘History of the *Australian Jewish News* in print and online’, in *Seventy Five Years: The History of a Jewish Newspaper*, The Australian Jewish Historical Society, Sydney, 1970.

48 Pinchas Goldhar, ‘The Press of the Jewish Community in Australia: Overview of the Yiddish-English and Yiddish Press of the Fifth Continent since 1842’, in Arthur S. Rose (Roz) and Israel (Yisrael) Sher (eds.), *Australian Yiddishe (Yiddish or Jewish) Almanac*, Melech Ravitch (ed.) (Melbourne: York Press, 1937), 258–74; Alan Crown , ‘The Jewish Press in Australia’, Arts: Journal of the Sydney University Arts Association, 15 (1990), 87–107 .

49

Gianfranco Cresciani , ‘The Second Awakening: The Italia Libera Movement’, Labour History, 30 (1976), 22–37 , at 30; Evan Smith , ‘Shifting Undesirability: Italian Migration, Political Activism and the Australian Authorities From the 1920s to the 1950s’, Immigrants & Minorities, 40, 1–2 (2022), 106–31.

50

Gianfranco Cresciani , Fascism, Anti-Fascism and Italians in Australia (Canberra: ANU Press, 1980), 34–5 & 43; Smith, *op. cit.* (note 38), 112–3.

51

Gaetano Rando , ‘Expressions of the Calabrian Diaspora in Calabrian Australian Writing’, in Gaetano Rando and Gerry Turcotte (eds.), Literary and Social Diasporas: An Italian Australian Perspective (Brussels: Peter Lang, 2007), 109–45, at 130; Carmelo Genovese , Alfredo Strano: An Italian Migrant Who Made a Difference (Perth: ANFE, 2011); Alfredo Strano , Luck Without Joy: A Portrayal of a Migrant, Elizabeth P. Burrows (trans.) (Fremantle, WA: Fremantle Press, 1986).

52 Marianna Piantavigna, ‘“We Know That the Press Is a Powerful Weapon”: Italian-Language Newspapers Defining and Representing the Italian Communities of Australia (1885–1972)’, PhD Thesis, University of Western Australia, 2021, 246–7. See also: Hilary Berthon, ‘A Treasure Trove of Community Language Newspapers’, in Dewhirst and Scully (eds.), *The Transnational Voices of Australia’s Migrant and Minority Press*, 209–24, at 217.

53 NAA: A434, 1950/3/30375, J.M. Allan, Officer-in-Charge, Tobacco Industry, Department of Agriculture, WA, to Under-Secretary, Department of Agriculture, WA, 30 June 1948. See: *Michelides: The Tobacco Pioneers: A Family History*, Vivid Publishing, 2015.

54

Osvaldo Bonutto , A Migrant’s Story: The Struggle and Success of an Italian-Australia, 1920s–1960s (Brisbane: H. Pole, 1963).

55 NAA: A434, 1950/3/30375, Heyes to H.C. Smith, Department for Lands and Immigration, Western Australia, 4 August 1948.

56 NAA: A434, 1950/3/30375, Chaffey to Frank W. Bulcock, Director-General of Agriculture, 17 September 1948; Heyes to Bulcock, 18 October 1948.

57 NAA: A434, 1950/3/30375, T.H. Strong, Acting Director, Bureau of Agricultural Economics, to E.J.B. Foxcraft, Department of Immigration, 14 July 1950.

58 NAA: A434, 1950/3/30375, Heyes to Director of Employment, 28 July 1950.

59 NAA: A434, 1950/3/30375, W. Funnell, Department of Labour and National Service, to Heyes, 30 August 1950.

60 NAA: A434, 1950/3/30375, confidential, note, Heyes, 26 October 1950; Heyes to Director, War Service Land Settlement Division, Department of the Interior, 16 January 1951.

61 NAA: A434, 1950/3/30375, Wilson to Heyes, 23 January 1951.

62 NAA: BP216/1, SS62, see the lists for 1952-1956; NAA: BP25/1, YUGOSLAVIAN SARAJLIC S, 1964–65.

63 Tyrrell, *op. cit.* (note 11), 113.

64

C. Sardu , A. Mereu , G. Pitzalis , L. Minerba and P. Contu , ‘Smoking Trends in Italy From 1950 to 2000’, Journal of Epidemiology and Community Health, 60, 9 (2006), 799–803 16905726 10.1136/jech.2005.043703PMC2566030.

65

Barbara Forey , Jan Hamling , John Hamling , Alison Thornton and Peter Lee , International Smoking Statistics: Greece (Sutton: PN Lee Statistics & Computing, 2015), 16 ; WHO, Gender and Tobacco in the Eastern Mediterranean Region (Cairo: WHO Regional Office for the Eastern Mediterranean, 2005); Fred Pampel , ‘Divergent Patterns of Smoking Across High-Income Nations’, in E.M. Crimmins , S.H. Preston and B. Cohen (eds.), International Differences in Mortality at Older Ages: Dimensions and Sources (Washington: National Academic Press, 2010), ch. 5 21977541.

66

Barbara Forey , Jan Hamling , John Hamling , Alison Thornton and Peter Lee , International Smoking Statistics: Comparisons Between Countries (Sutton: PN Lee Statistics & Computing, 2016), 23 ; Marie Ng , Michael K. Freeman , Thomas D. Fleming , Margaret Robinson , Laura Dwyer-Lindgren , Blake Thomson , Alexandra Wollum , Ella Sanman , Sarah Wulf , Alan D. Lopez , Christopher J.L. Murray and Emmanuela Gakidou , ‘Smoking Prevalence and Cigarette Consumption in 187 Countries, 1980–2012’, JAMA, 311, 2 (2014), 183–9224399557 10.1001/jama.2013.284692; C. Hill , ‘Trends in Tobacco Use in Europe’, Journal of the National Cancer Institute Monographs, 12 (1992), 21–41616805.

67 ‘Advertising’, *To Ethnico Vema*, Arncliffe NSW, 3 February 1937, 4.

68 ‘Advertising’, *To Ethnico Vema*, Arncliffe NSW, 22 June 1949, 4; ‘Advertising’, *To Ethnico Vema*, Arncliffe NSW, 8 March 1950, 4.

69 ‘Kookaburra Cigarette Paper Tubes’, *Hebrew Standard of Australasia*, Sydney, 17 November 1933, 5.

70 ‘Advertising’, *Australian Jewish News*, Melbourne, 12 October 1951, 9.

71 ‘“Levy’s Own” Quality Tobacco’, *Hebrew Standard of Australasia*, Sydney, 3 October 1940, 5; Glass Negative – George Street Sydney, circa 1912, City of Sydney Archives, A-01000363; ‘The Local Shop Is Alive, so Stick That in Your Pipe and Smoke It’, *Sydney Morning Herald*, 22 April 2002; Su-Lin Tan, ‘Historical Sol Levy Sells for $6.7m’, *Financial Review*, Sydney, 10 October 2014.

72 ‘Golden Key Cigars’, *Hebrew Standard of Australasia*, Sydney, 15 October 1915, 12.

73 Glass Negative – George Street Sydney, circa 1912, City of Sydney Archives, A-01000363: https://archives.cityofsydney.nsw.gov.au/nodes/view/1725791.

74 ‘Advertising’, *To Ethnico Vema*, Arncliffe NSW, 1 February 1950, 8.

75 ‘Advertising’, *Australian Jewish News*, Melbourne, 6 May 1955, 9.

76 ‘Advertising’, *To Ethnico Vema*, Arncliffe NSW, 29 May 1935, 2.

77 ‘Greek Bid for Tobacco Market’, *To Ethnico Vema*, 31 May 1939, 7.

78 ‘Greece To-Day: Paper Industry in Greece’, *To Ethnico Vema*, Arncliffe NSW, 13 October 1948, 1.

79 ‘Statement of Receipts and Expenditure Covering the Greek Sub Branch Fete’, *To Ethnico Vema*, Arncliffe NSW, 20 July 1947, 6.

80 ‘The Cigarette Originating in Syria 102 Years Ago’, *To Ethnico Vema*, Arncliffe NSW, 5 June 1935, 1.

81 ‘Radio Programme “For New Australians”’, *To Ethnico Vema*, Arncliffe NSW, 27 January 1954, 3.

82 ‘Radio Programme “For New Australians”’, *To Ethnico Vema*, Arncliffe NSW, 7 January 1953, 2.

83 ‘The Beer Lover’s Order’, *To Ethnico Vema*, Arncliffe NSW, 7 November 1934, 1.

84
*English for Newcomers to Australia*, R.G. Crossley (consultant), 1st edn. (Sydney: Commonwealth Office of Education, July 1948), Lesson 1(a), 9.

85 ‘Migrants’ Tuition “Practical”’, *News*, Adelaide, 31 July 1950, 16.

86 NAA: MP742/1, 264/4/209; NAA: C605, LA192A; NAA: C605, LA192B; NAA: C605, LA192C; MP148/2, 1941/1614; MP742/1, 264/4/209.

87 NAA: A1539, 1944/W/191.

88 NAA: D1975, Z1944/83; MP742/1, 255/5/48.

89 ‘Non si possono avere sigarette? (Can’t Have Cigarettes?)’, *Il Risveglio*, Sydney, 7 February 1945, 3.

90 Luigi Gussago, ‘Terra Recognita: Early Italian Farmers in Eastern Australia (1820–1950)’, *Altreiltalie*, 66 (2023), 51–71; Laura Faggion and Raffaello Furlan, ‘Post-WWII Italian Migration From Veneto (Italy) to Australia and Transnational Houses in Queensland’, *International Journal of Arts*, 6, 1 (2016), 1–10; Bruce Missingham, Jacqui Dibden and Chris Cocklin, ‘A Multicultural Countryside? Ethnic Minorities in Rural Australia’, *Rural Society*, 16, 2 (2006), 131–50.

91 Bonutto, *op. cit.* (note 54), 10.

92 ‘Farm in vendita a Wyemo (Yearbook) Qld. (Farm for sale in Wyemo (Yelarbon) Qld.)’, *Il Risveglio*, Sydney, 23 July 1947, 4.

93 ‘“Il Tabacco E’ All Right … Ma …” (“Tobacco Is All Right … but …”)’, *Il Risveglio*, Sydney, 26 June 1946, 3.

94 ‘Lo sviluppo dell’organizza zione cooperativa fra i coltivatori di tabacco nel North Queensland (the Development of Cooperative Organization Among Tobacco Farmers in North Queensland)’, *Il Risveglio*, Sydney, 17 September 1947, 2.

95 ‘Protesta per l’aumento del tobacco e delle sigarette (Protests due to the increase in tobacco and cigarettes)’, *Il Risveglio*, Sydney, 19 February 1947, 2.

96

Alison Reid , Enzo Merler , Susan Peters , Nimashi Jayasinghe , Vittoria Bressan , Peter Franklin , Fraser Brims , Nicholas H. de Klerk and Arthur W. Musk , ‘Migration and Work in Postwar Australia’, Occupational & Environmental Medicine, 75, 1 (2018), 29–36 28756413 10.1136/oemed-2017-104322, at 31.

97 ‘Australian Relief for Italy’, *Il Risveglio*, Sydney, 9 April 1946, 2.

98 ‘Advertising’, *To Ethnico Vema*, Arncliffe NSW, 3 August 1932, 3.

99 ‘Michelides Ltd. Tobacco and Cigarettes’, *West Australian*, Perth, 20 May 1935, 6; ‘Best Tobacco in World: Grown in Westralia’, *Richmond River Herald & Northern Districts Advertiser*, Coraki NSW, 13 August 1935, 4.

100 ‘Advertising’, *Stampa Italiana*, Perth, 26 August 1932, 3.

101 Cox, *The Global Cigarette*, 47.

102 ‘Perth Tobacco Industry: Perth Factory Extension’, *To Ethnico Vema*, Arncliffe NSW, 31 January 1934, 4; ‘Tragedy in Tobacco: Australian Industry Crippled Manufacturers’ Protests’, *To Ethnico Vema*, Arncliffe NSW, 30 March 1932, 3; ‘Annual Report and Balance Sheet of Michelides Ltd’, *To Ethnico Vema*, Arncliffe NSW, 7 October 1936, 5.

103

Yiannis E. Dimitreas , Transplanting the Agora: Hellenic Settlement in Australia (St Leonards NSW: Allen & Unwin, 1998), 187 .

104 NAA: F1, 1952/853, 1952-56. This was alongside William Butler Tobacco Co., The Nelson Tobacco Company, Seale & Smale Ltd., Associated Tobacco Manufacturers Ltd., F.H.V. Tobacco Company, Louis Carsten & Company, REMA Tobacco Co., The General Tobacco Trading Company, Ascott Henderson Pty. Ltd., Thomas Brown & Sons, A.I. Risdale, M.M. Toglini (Tognolini), Grumould Pty. Ltd., North Queensland Tobacco Growers Co-Op, Mil Tobacco Co., Metro Tobacco Manufacturers, A.E. Stower, & Alan Palmer & Company.

105 Tobacco Supplies to Aborigines, 23 September 1944, NAA: F1, 1952/853, 1952–56.

106
*Michelides*, 256–70.

107 ‘Tobacco ‘Major Problem’: Migrants Make Distribution More Difficult’, *Courier-Mail*, Brisbane, 18 February 1950, 5; ‘Migrants Mean More Smokers’, *Morning Bulleting*, Rockhampton QLD, 18 February 1950, 5; ‘Tobacco Supply Major Problem’, *Maryborough Chronicle*, Maryborough QLD, 18 February 1950, 4; ‘Migrants Need Tobacco Too’, *Townsville Daily Bulletin*, Townsville QLD, 18 February 1950, 1.

108 ‘Worry Blamed for Smoking’, *Sunday Mail*, Brisbane, 22 January 1950, 5.

109 NAA: 12700, 1196, 1946.

110 ‘Plenty of Cigarettes’, *Sun*, Sydney, 30 April 1950, 5; ‘Balts Sell Cigarettes’, *Daily News*, Perth, 20 May 1948, 4.

111 NAA: A436, 49/5/269, 1947–49.

112 The Greek-language newspaper *To Ethnico Vema* (*National Tribune*) is a good example of this: ‘Προσέχετε την Υγείαν (Taking Care of Health/Health Care)’, *To Ethnico Vema*, Arncliffe NSW, 18 March 1931, 7; ‘Θεραπεία του Καρκίνου (Cancer Therapy)’, *To Ethnico Vema*, Arncliffe NSW, 13 February 1935, 7; ‘Προς Καταπολέμησιν του Καρκίνου (The Fight Against Cancer)’, *To Ethnico Vema*, Arncliffe NSW, 15 May 1935, 4; ‘Άδικοι θάνατοι από καρκίνον του δέρματος (Unjust Deaths From Skin Cancer)’, *To Ethnico Vema*, Arncliffe NSW, 22 May 1935, 6; ‘Ο Ανικατάστατης του ράδιου (The Substitute of Radiotherapy)’, *To Ethnico Vema*, Arncliffe NSW, 19 February 1936, 6.

113 ‘I Morti Per Il Cancro In Australia (Cancer Deaths in Australia)’, *Stampa Italiana*, Perth, 12 February 1932, 2; ‘Un Dispensario Per La Cura Del Cancro (A Dispensary for the Treatment of Cancer)’, *Stampa Italiana*, Perth, 7 January 1932, 3; ‘Notizie in Fascio (News in a Bundle)’, *Stampa Italiana*, Perth, 7 January 1932 & 8 March 1932, 2; ‘Contro Il Cancro (Against Cancer)’, *Il Giornale Italiano*, Sydney, 20 December 1933, 3; ‘La nature du sol el le cancer (The Nature of the Soil and Cancer)’, *Le Courrier Australien*, Sydney, 16 December 1932, 3. *Le Courrier Australien* had interestingly predicted that cancer would become a ‘historical curiosity’ by the year 2,000: ‘En l’an 2,000 (In the year 2,000)’, *Le Courrier Australien*, Sydney, 8 December 1933, 3.

114 ‘Τα Αιτία Του Καρκίνου (The Causes of Cancer)’, *To Ethnico Vema*, Arncliffe NSW, 20 September 1936, 3; ‘Ο Καρκίνος: Αύξησις Θνησιμότητος (Cancer: Increased Mortality)’, *To Ethnico Vema*, Arncliffe NSW, 14 April 1937, 5; ‘Αντικαρκινός Πόλεμος: Ανέγερσις περίπτερου παρά τω νοσοκομείω “Prince Henry” (War on Cancer: Erection of a Pavilion in Front of the “Prince Henry” Hospital)’ & ‘Ο Καρκίνος ως Δευτέρα μεγάλη μάστιγξ (Cancer the Second Great Scourge)’, *To Ethnico Vema*¸ Arncliffe NSW, 13 October 1937, 6.

115 ‘Card Party Will Assist Anti-Cancer Appeal’, *Australian Jewish Herald*, Melbourne, 17 June 1937, 5; ‘Anti-Cancer Appeal’, *Age*, Melbourne, 15 June 1937, 5.

116 ‘Jews and the Fight Against Cancer’, *Hebrew Standard of Australasia*, Sydney, 10 December 1936, 2.

117 ‘Cancer Research Scholarship at Hebrew University’, *Hebrew Standard of Australasia*, Sydney, 1 May 1941, 10.

118 To only name a few: ‘Ο Καρκίνος εν Αγγλία (Cancer in England)’, *To Ethnico Vema*, Arncliffe NSW, 14 December 1938, 5; ‘Αίτια των Θάνατων (Causes of Death)’, *To Ethnico Vema*, Arncliffe NSW, 12 January 1938, 5; ‘Πόθεν προέρχεται ό καρκίνος (Where Does Cancer Come From?)’, *To Ethnico Vema*, Arncliffe NSW, 12 October 1938, 8; ‘Ανεκάλυφθη η Αιτιολόγια του Καρκίνου; (The Causes of Cancer Discovered?)’, *To Ethnico Vema*, Arncliffe NSW, 10 May 1939, 3; ‘Il Dott. Koch Esperimentera’ in Australia (Dr Koch Will Experiment in Australia)’, *Il Giornale Italiano*, Sydney, 14 December 1938; ‘La Lotta Contro Il Cancro in America (The Fight Against Cancer in America)’, *Il Giornale Italiano*, Sydney, 15 March 1939, 6; ‘Enquete (Investigation)’, *Le Courrier Australien*, Sydney, 1 September 1939, 2; ‘L’Institut du cancer à Strasbourg (The Cancer Institute in Strasbourge)’, *Le Courrier Australien*, Sydney, 7 April 1939, 4.

119 ‘The Fight Against Cancer’, *Australian Jewish Herald*, Melbourne, 31 October 1940, 12.

120 ‘Έγκαιρος διαγνώσις και θερπεία του καρκίνου (Early Diagnosis and Treatment of Cancer)’, *To Ethnico Vema*¸ Arncliffe NSW, 8 October 1941, 3.

121 For example, see: ‘Ο Καρκίνος (Cancer)’, *To Ethnico Vema*¸ Arncliffe NSW, 15 December 1943, 8; ‘Νεώτερα θεραπεία του καρκίνου προστάτου (New Treatment for Prostate Cancer)’, *To Ethnico Vema*, Arncliffe NSW, 18 July 1945, 3; ‘Εύκολος θεραπεία του καρκίνου (Easy Treatment for Cancer)’, *To Ethnico Vema*, Arncliffe NSW, 24 May 1844, 3.

122 ‘The Treatment of Cancer: Advice by the Anti-Cancer Council of Victoria’, *Sydney Jewish News*, Sydney, 3 May 1936, 5. See also: ‘Anti-Cancer Campaign’, *Sydney Jewish News*, Sydney, 29 March 1946, 4; ‘New Drive Against Cancer: Advice to the Public of the State by the Anti-Cancer Council in Victoria’, *Sydney Jewish News*, Sydney, 22 March 1946, 5.

123 ‘Publication of Free Handbook’, *Sydney Jewish News*, Sydney, 22 March 1946, 6; ‘Early Treatment Essential Says Council’, *Australian Jewish Herald*, Melbourne, 5 April 1946, 5.

124 ‘Θεραπεύεται εύκολος ο καρκίνος του δερματός, των χείλων και του μάστου (Easy Treatment of Skin, Lips and Breast Cancer)’, *To Ethnico Vema*¸ Arncliffe NSW, 3 April 1946, 3; ‘Heartening Anti-Cancer Discovery’, *Hebrew Standard of Australasia*, Sydney, 20 March 1947, 9.

125 ‘British Cancer Campaign’, *Le Courrier Australien*, Sydney, 11 October 1946, 7.

126 Rene Sudre, ‘French Cancer Research’, *Le Courrier Australien*, Sydney, 11 October 1946, 7. See also: ‘L’Institut du Radium’, *Le Courrier Australien*, Sydney, 30 November 1945, 8.

127 ‘Medicina: L’Insidia Del Cancro (Medicine: The Risk of Cancer)’, *Il Risveglio*, Sydney, 7 April 1948, 2; ‘Συνέστηθη μια ειδική οργανώσις προς καταπολέμησιν του καρκίνου (A Special Organisation Founded to Fight Cancer)’, *To Ethnico Vema*, Arncliffe NSW, 6 November 1946, 3.

128 ‘Great Discovery by One Italian Doctor’, *Il Risveglio*, Sydney, 3 September 1947, 4.

129 ‘Το πρόβλημα του καρκίνου θα λυθή επί των ημερών μας (The Problem of Cancer Will Be Solved in Our Days)’, *To Ethnico Vema*¸ Arncliffe NSW, 15 August 1951, 3; Νεώτεραι ελπίδες θεραπείας του καρκίνου (New Hopeful Therapies for Cancer)’, *To Ethnico Vema*, Arncliffe NSW, 2 May 1951, 3.

130 ‘Η ατομική ενέργεια εις τον πόλεμον κατά του καρκίνου (Atomic Energy in the War Against Cancer)’, *To Ethnico Vema*, Arncliffe NSW, 9 January 1952, 5.

131 ‘Grant for Cancer Research’, *Hebrew Standard of Australasia*, Sydney, 13 March 1953, 5.

132 ‘Περί την μεγάλην μάστιγα: Ο καρκίνος και η κρεβιόζη (About the Great Plague: Cancer and Thrombosis)’, *To Ethnico Vema*, Arncliffe NSW, 6 February 1952, 3; ‘Τι λέει Βιεννέζος Καθητής (What a Viennese Doctor says)’, *To Ethnico Vema*, Arncliffe NSW, 19 March 1952, 5; ‘Μια ιατρός πειραματίζεται με τον καρκίνον εφ’ εαυτής! (A Doctor Experiments With Cancer Themselves!)’, *To Ethnico Vema*, Arncliffe NSW, 26 March 1952, 3; ‘Η μάχη κάτα του καρκίνου (The Battle Against Cancer)’, *To Ethnico Vema*, Arncliffe NSW, 26 March 1952, 6.

133 ‘Μεγάλαι αι ελπίδες δια την καταπολέμησιν του καρκίνου (High Hopes for the Fight Against Cancer)’, *To Ethnico Vema*, Arncliffe NSW, 2 July 1952, 5. Quote translated from Greek to English by the authors.

134 ‘Ο καρκίνος και η λευχαμία θα θεραπεύονται (Cancer and Leukemia Will Be Cured)’, *To Ethnico Vema*, Arncliffe NSW, 4 February 1953, 3.

135 ‘Το μεγαλήτερον μυστηριον της ιατρικής (The Greatest Mystery of Medicine)’, *To Ethnico Vema*, Arncliffe NSW, 27 October 1954, 6.

136 ‘Ο άνθρωπος που ενίκησε με την πιστίν του τον καρκίνον (The Man Who Conquered Cancer With His Faith)’, *To Ethnico Vema*, Arncliffe NSW, 17 November 1954, 3.

137 ‘Le Cause Del Cancro (The Cause of Cancer)’, *Il Risveglio*, Sydney, 22 January 1955, 2.

138 ‘For Hospital, Anti-Cancer Campaign’, *Australian Jewish News*, Melbourne, 9 May 1958, 11.

139 ‘Soccer for Anti-Cancer Campaign’, *Australian Jewish News*, Melbourne, 13 June 1958, 14.

140 See: Simon Chapman and Melanie Wakefield , ‘Tobacco Control Advocacy in Australia: Reflection on 30 Years of Progress’, Health Education & Behaviour, 28, 3 (2001), 274–8910.1177/10901981010280030311380049.

141 Tyrrell, *op. cit.* (note 11), 193-5; Berridge, ‘The Policy Response to the Smoking and Lung Cancer Connection in the 1950s and 1960s’, 1185-209; Toth-Peter et al., *op. cit.* (note 12), 1.

142 Robert Imray to Prime Minister SM Bruce, Greymare, South West Line, Queensland, 10 March 1928, NAA: A1928, 1099/5, 146387.

143 ‘Οι Κίδυνοι του Καπνού (The Dangers of Smoking)’, *To Ethnico Vema*, Arncliffe NSW, 10 May 1933, 8; ‘Le Lettere Del Nostri Lettori: Concordia e Tobacco (Letters From Our Readers: Concordia and Tobacco)’, *Il Giornale Italiano*, Sydney, 6 December 1933, 2. See also: ‘Sous le “rayon” miraculeux (Under the Miraculous “Ray”)’, *Le Petit Marseillais*, Marseille, 17 January 1933, 3; ‘Le professeur Imbert a parle, hier de ce terrible fleau: le cancer (Professor Imbert Spoke Yesterday About This Terrible Scourge: Cancer)’, *Le Petit Provençal*, Marseille, 27 January 1935, 3.

144 Theatres and Public Halls Act, No. 13, Parliament of New South Wales, Sydney, 1908.

145 ‘Letter to the Editor: Smoking in a Public Hall’, *Hebrew Standard of Australasia*, Sydney, 15 August 1946, 4.

146 ‘Alcuni Fatti D’Igiene e Medicina (Some Facts of Hygiene and Medicine)’, *Il Risveglio*, Sydney, 19 February 1947, 4.

147 ‘Alcuni Fatti D’Igiene e Medicina (Some Facts of Hygiene and Medicine)’, *Il Risveglio*, Sydney, 19 February 1947, 4.; ‘Harold J. Stewart, Medical Professor’, *New York Times*, New York, 5 April 1975, 32.

148 ‘Κάπνισμα και Καρκίνος (Smoking and Cancer)’, *To Ethnico Vema*, Arncliffe NSW, 27 August 1952, 3.

149 ‘Τον καρκίνον του πνεύμονος προκαλούν αι αναθυμιάσεις της ασφάλτου και το τσιγάρο! (Lung Cancer Is Caused by Asphalt Fumes and Cigarettes!)’, *To Ethnico Vema*, Arncliffe NSW, 10 December 1952, 7.

150 “Ελβετος ιατρός έφευρε το “Αντικαρκίνον” σιγαρέττον (Swiss Doctor Invents the Anti-Cancer Cigarettes)’, *To Ethnico Vema*, Arncliffe NSW, 2 June 1954, 6.

151 ‘Per Questi Il Fumo E’ Un Serio Pericolo (For These Smoking Is a Serious Danger)’, *Il Risveglio*, Sydney, 1 December 1952, 4.

152 ‘Στο εδώλιο του κατηγορούμενου το τσιγάρο, τα ποτά και οι καφέδες (In Defence of Cigarettes, Drinks and Coffee)’, *To Ethnico Vema*, Arncliffe NSW, 15 July 1953, 3.

153 ‘Αποτέλει το κάπνισμα νοσογόνον παράγοντα καρκίνου πνευμονικού; (Is Smoking a Causative Agent of Lung Cancer?)’, *To Ethnico Vema*, Arncliffe NSW, 3 June 1953, 6.

154

Richard Doll, A. Bradford Hall , H. Binysh and Lennox Johnston , ‘Smoking and Lung Cancer’, British Medical Journal, 1, 4808 (1953), 505–710.1136/bmj.1.4808.505PMC201541813009269.

155 Donald G. Cooley, ‘Who Says Smoking Causes Lung Cancer?’, *True: The Man’s Magazine*, July 1954, 84–7, at 86.

156 ‘Ο Καρκίνος των πνευμόνων (Lung Cancer)’, *To Ethnico Vema*, Arncliffe NSW, 14 April 1954, 5.

157 ‘Με Δυό Λόγια (In Brief)’, *To Ethnico Vema*, 24 November 1954, 1.

158 ‘Οι ‘Αγγλοι, υο κάπνισμα και ο καρκίνος του πνεύμονος (The English, Smoking and Lung Cancer)’, *Elefthera Phoni*, Perth, 21 February 1957, 4.

159 ‘Οι ‘Αγγλοι, υο κάπνισμα και ο καρκίνος του πνεύμονος (The English, Smoking and Lung Cancer)’, *Elefthera Phoni*, Perth, 21 February 1957, 4.

160 ‘Σύντομα νέα (Soon to Be News)’, *Elefthera Phoni*, Perth, 7 March 1957, 6.

161 ‘Smoking and Lung Cancer’, *Australian Jewish News*, Melbourne, 13 September 1957, 13.

162 ‘Roundabout With Pamela Ruskin: Power of the Press’, *Australian Jewish News*, Melbourne, 8 June 1956, 5.

163 ‘Αντιβιοτικόν κατά του καρκίνου: Αναμφίβολος η σχέσις καρκίνου – καπνίσματος (Antibiotic Against Cancer: The Cancer-Smoking Relationship Is Undoubted’, *Neos Kosmos*, Melbourne, 10 April 1957, 3.

164 ‘Νέα απ’ όλο τον κόσμο (News From All Over the World), *Neos Kosmos*, 28 March 1962, 6; ‘Τσιγάρο: Αργός θάνατος (Cigarettes: A Slow Death)’, *Neos Kosmos*, Melbourne, 1 August 1962, 8.

165 NAA: A4940, C3118, confidential, Cabinet Minute, Decision 835, 17 May 1960.

166 NAA: A4940, C3118, confidential, Cabinet Minute, Decision 637, 5 February 1963.

167
*Op. cit.* (note 10).

168 NAA: A4940, C3118, confidential, memorandum, Attorney-General, Garfield Barwick, to Minister for Health, H.W. Wade, 24 December 1962.

169 NAA: A4940, C3118, confidential, memorandum, Minister for Primary Industry, C.F. Aderman, to Minister for Health, H.W. Wade, undated.

170 NAA: A4940, C3118, confidential, memorandum, Minister for Health, H.W. Wade, to Cabinet, 30 January 1963.

171 NAA: A4940, C3118, confidential, Notes on the Cabinet Discussion, 1 February 1963.

172 NAA: A4940, C3118, confidential, Cabinet Minute, Decision 637, 5 February 1963.

## Long descriptions

### Table 1. Long description

The table consists of five columns: Country or Colony, 1933, 1947, 1954, and 1961.

* Italy: 26,756 in 1933; 33,632 in 1947; 119,897 in 1954; 228,296 in 1961.

* Greece: 8,337 in 1933; 12,291 in 1947; 25,862 in 1954; 77,333 in 1961.

* France: 2,587 in 1933; 2,215 in 1947; 4,699 in 1954; 5,409 in 1961.

* Egypt: 561 in 1933; 803 in 1947; 8,150 in 1954; 16,287 in 1961.

* Cyprus: 502 in 1933; 681 in 1947; 5,773 in 1954; 8,576 in 1961.

* Jewish (by religion): 23,555 in 1933; 32,019 in 1947; 48,436 in 1954; 59,329 in 1961.

Navigate back to Table 1..

### Figure 3. Long description

The advertisement is enclosed in a decorative Greek key border.

At the top, large bold Greek text reads SIGARETTA PAPASTRATOU, followed by a paragraph in Greek introducing the Michaelides Tobacco Company as the exclusive representative of Papastratos for the Greek-Australian public.

The center contains two graphic panels. The upper panel shows a dark background with a white classical fluted column on the right and a circular object resembling a globe or sun in the center. The lower panel features a large stylized number 1 overlapping a dark circle, with the word Hellas printed to the right and PAPASTRATOS ATHENS in small print below.

Below the graphics, a line of Greek text encourages supporting the Greek National Economy. This is followed by bold English text stating Smoke the World Famous HELLAS Cigarettes.

At the bottom, a Note section in English explains the company policy of maintaining absolute uniformity in tobacco blends to ensure quality never varies. The ad concludes with the signature Papastratos Cigarette Manufacturing Co. A T H E N S.

Navigate back to Figure 3..

### Figure 4. Long description

A rectangular newspaper advertisement with a decorative border.

* The headline at the top reads Levy's Own Quality Tobacco in a bold serif font.

* The body text states that the tobacco is available at 7 forward slash - per pound and is honestly worth 10 forward slash - pound.

* It also lists BESTEVER Flake Cut for the Pipe at 8 forward slash - per pound and FLAKE FINE CUT for Cigarette worth 12 forward slash - per pound.

* A call to action follows: Send 6d. in stamps for liberal sample.

* Additional products listed include Zig-Zag Papers, 60 leaves pkt, 2 forward slash 6 doz and Zig-Zag Cigarette-making Machine, 1 forward slash -.

* The bottom section provides contact details: Call or write to: SOL LEVY, Established 38 years, 713 GEORGE STREET, SYDNEY.

* The final line reads: Goods sent C.O.D. if desired.

Navigate back to Figure 4..

### Figure 5. Long description

The street scene is anchored by a row of multi-story commercial buildings.

On the far left, a tall brick building features arched windows and decorative ironwork over the sidewalk. In front, a horse-drawn cart stands near a group of men in suits and hats.

Moving right, the central building is Sol Levy Tobacconist. The facade is heavily branded with signs for Yankee Doodle and Capstan Tobacco and Cigarettes. The ground floor appears to be under renovation or construction, with exposed timber beams and workers standing near the entrance.

To the right of the tobacconist, a large curved billboard above the awning reads Smoke Wills's Vice-Regal Mixture. Below this, another horse-drawn cart is parked, and a shop window displays various goods.

The far right building is the Emporium Coffee Palace, with a sign for George D. Gal above it.

The foreground shows a wide, dusty street with visible tram tracks running horizontally. Pedestrians are scattered along the sidewalk under the continuous decorative metal awnings that shade the storefronts.

Navigate back to Figure 5..

### Figure 7. Long description

The advertisement is enclosed in a decorative geometric border. At the top right, a detailed line drawing shows a rectangular, vertically ribbed metal cigarette lighter with its top cap flipped open and a small flame visible. To the left of the lighter is a white rectangular logo containing a small candle icon above the word Upson in cursive script, followed by the block text FINGER TIP CIGARETTE LIGHTER. Below the illustration is a paragraph of Greek text. At the bottom, the text switches back to English, identifying the Sole Distributors as T. R. URBAN & CO. located at 247, Elizabeth St., Sydney.

Navigate back to Figure 7..

### Figure 8. Long description

A black and white print advertisement divided into two main sections.

At the top left, a line drawing shows the dispenser clamped to a car's steering column. To the right of the drawing, the price 67 forward slash 6 is printed in large bold numbers, followed by the product name REDILITE CIGARETTE DISPENSER in all caps.

Below the header, a block of text reads: HANDS YOU A LIGHTED CIGARETTE WHILE YOU DRIVE. Holds 25 cigarettes — Dependable — Safe — Foolproof. Available to suit 6 or 12 Volt Batteries. Walnut or Mahogany finish. Connects to ammeter. Self clamps to steering column in 3 minutes.

The bottom section is a dark rectangular block containing the company logo. On the left is a triangular emblem with a stylized V and the words VICTORIA SEAT COVERS. To the right, the word VICTORIA is printed in large, bold, white sans-serif letters. Below this, in smaller text, it reads CAR SERVICES P T Y dot L T D dot.

Navigate back to Figure 8..

### Figure 9. Long description

A vertical print advertisement divided into several sections.

* Top section: A halftone illustration of a man in formal attire sitting at a table, using a knife and fork to eat from a plate. The illustration is set within a tilted rectangular frame.

* Center section: A large, dark downward-pointing arrow overlaps the illustration. Inside the arrow, white text reads IT AIDS DIGESTION. Below this, in a script font, it says Try it after every meal!

* Middle-left section: A block of justified text begins with a large decorative W. The text reads: Wrigley's chewing gum is the perfect finish to every meal, for it assists digestion. Its clean mint flavour leaves the mouth tasting fresh, its pleasant fragrance sweetens the breath. Wrigley's makes that after-meal cigarette taste better, too. Remember to keep some always on hand. You'll like it!

* Middle-right section: Two rectangular gum packets are shown in a three-dimensional perspective. The top packet is labeled WRIGLEY'S P. K. CHEWING SWEET. The bottom packet is labeled WRIGLEY'S SPEARMINT CHEWING SWEET. Small text below them reads: Also Spearmint in six bar packets, 3d.

* Bottom section: Large, bold sans-serif text across the base reads: TAKE YOUR CHANGE IN WRIGLEY'S.

Navigate back to Figure 9..

### Figure 10. Long description

The photo captures a two-story Edwardian-style building on a street corner.

* At the top, several power lines cut across a clear sky. Large signage for BOANS L T D is visible on a taller building section in the background.

* The main building facade features a curved corner with decorative masonry and a series of rectangular windows on the second floor. A dark horizontal band between the floors displays the text BRAHAM BROS.

* At the street level, a continuous white awning wraps around the building. Underneath, the shopfront for Michelides Tobacconists is visible with large glass windows.

* The foreground shows a wide, paved intersection. Pedestrians in mid-century attire, including men in suits and hats, walk across the street.

* To the right, several vintage automobiles are parked along the curb, and a group of people gathers near a street stall or entrance.

* On the far left, a partial view of a large sign reads ANS.

Navigate back to Figure 10..

### Figure 11. Long description

A vertical print advertisement organized into four main sections.

* Top Section: The headline reads Sunny WESTERN AUSTRALIA GIVES AUSTRALIA THIS REALLY FINE TOBACCO! in bold, capitalized letters. Behind the text is a stylized rising sun with radiating lines over a horizon line.

* Central Section: A rectangular tin of White Oak Tobacco is angled in the center. The tin label includes an oak tree logo and the text WHITE OAK TOBACCO. Below the tin, cursive text reads - and what a reception Australia gives it!

* Body Text Section: A paragraph of small print describes the tobacco as bright, clear, and golden-hued. Pricing is listed as 1 oz tin 9d and 2 oz tin 1 forward slash.

* Footer Section: The bottom features the brand name WHITE OAK in large block letters with an oak tree icon. Below this, it states HIGH GRADE FINE CUT TOBACCO and Made by MICHELIDES L T D, PERTH, W A dot.

Navigate back to Figure 11..

### Figure 12. Long description

The advertisement is structured as follows.

* Top Header: Two lines of Greek text in bold and standard weights. The first line reads SMOKERS! Watch your health! The second line warns about protecting the throat and chest from the harmful effects of bad tobacco.

* Central Left: A rectangular tobacco tin with rounded corners. The tin features the brand name LUXOR in large, stylized white serif letters at the top. Below this is a central circular emblem with a star and the initialism M L. Flanking the emblem are the words FINE and CUT. At the bottom of the tin, the words VIRGINIAN TOBACCO appear in a clean sans-serif font.

* Central Right: Several paragraphs of Greek text promoting the quality and value of Luxor tobacco. A specific note mentions that each box of LUXOR TOBACCO includes a free packet of fine French cigarette papers.

* Bottom Section: The advertisement concludes with a footer in Greek and English. It identifies the product as a result of the Greek-Australian Tobacco Industry. The manufacturer's name is printed in large, bold capital letters as MICHELIDES LIMITED, followed by the location PERTH W dot A dot.

Navigate back to Figure 12..

### Figure 13. Long description

A black and white print advertisement organized into three vertical sections.

* Top section: Greek text in bold capital letters states that this excellent tobacco costs only 9 d per ounce. The price 9 d is large and positioned in the top right corner.

* Middle section: A rectangular tin and a cylindrical open container of White Oak tobacco are shown side-by-side. The tin features a central oak tree logo and the words WHITE OAK FINE TOBACCO. Below the products, two lines of Greek text describe it as the best and cheapest tobacco at 9 pence for a 1 ounce tin, inviting customers to try it and be captivated by the quality.

* Bottom section: A large black banner contains the brand name WHITE OAK in white serif lettering, separated by an oak tree emblem. Below this, the text FINE CUT TOBACCO appears in bold. The final lines identify the product as a result of the Anglo-Hellenic Company, MICHELIDES LTD. - PERTH.

Navigate back to Figure 13..

### Figure 14. Long description

The advertisement is divided into two horizontal sections by a decorative border.

Top Panel:

Features a large, dark rectangular tin for Luxor Fine Cut Virginian Tobacco on the left. The tin has a central crest and bold white lettering. To the right of the tin, several paragraphs of Greek text promote the quality of the tobacco. Below the tin and text, the company name MICHELIDES LIMITED is printed in large block letters, followed by PERTH W dot A dot in a smaller font.

Bottom Panel:

This section advertises Alpha Paper and other cigarette paper brands. It contains multiple small rectangular logos and product labels arranged in a grid-like fashion.

- Top left: A label for Super Rice Paper.

- Top right: A label for Isis Pure Rice Paper.

- Center: A circular logo for Alpha Paper surrounded by Greek text.

- Bottom left: A label for Superior Quality papers.

- Bottom right: A label featuring a small illustration of a camel and the name S dot TOPAL SAVAS.

At the very bottom, contact information is listed for various Australian cities including MELBOURNE, SYDNEY, BRISBANE, and PERTH. The text throughout the bottom panel is a mix of English phrases like ASK FOR AUSTRALIAN MANUFACTURED CIGARETTE PAPERS and Greek descriptions.

Navigate back to Figure 14..

### Figure 15. Long description

The clipping is divided into two distinct rectangular advertisement blocks.

On the left is the White Oak tobacco advertisement. At the top, it reads FUMATE E RISPARMIATE! followed by the brand name WHITE in large, stylized white block letters on a black background. Below this is a small illustration of an oak tree. The word OAK appears in similar large block lettering. Underneath, it states FINE CUT TOBACCO. Smaller Italian text at the bottom describes the tobacco quality and lists the price as 1 forward slash 7 for 2 O Z.

On the right are two smaller stacked boxes. The top box contains Italian text regarding savings. The larger box below is for L. MISTRORIGO'S PHARMACY. The headline asks LA VOSTRA SALUTE E’ IN PERICOLO? followed by Scrivete alla farmacia italiana di Perth. The address is listed as 208 William Street, Perth (Western Australia). The text identifies Dott. L. Mistrorigo as a Chimico Italiano and provides a paragraph in Italian regarding medical specialties and a free catalog.

Navigate back to Figure 15..

